# Two UGT84 Family Glycosyltransferases Catalyze a Critical Reaction of Hydrolyzable Tannin Biosynthesis in Pomegranate (*Punica granatum*)

**DOI:** 10.1371/journal.pone.0156319

**Published:** 2016-05-26

**Authors:** Nadia N. Ono, Xiaoqiong Qin, Alexander E. Wilson, Gang Li, Li Tian

**Affiliations:** 1 Department of Plant Sciences, University of California Davis, Davis, California, United States of America; 2 Shanghai Key Laboratory of Plant Functional Genomics and Resources, Shanghai Chenshan Botanical Garden, Shanghai, China; 3 Shanghai Chenshan Plant Science Research Center, Chinese Academy of Sciences, Shanghai, China; Zhejiang University, CHINA

## Abstract

Hydrolyzable tannins (HTs) play important roles in plant herbivore deterrence and promotion of human health. A critical step in HT production is the formation of 1-*O*-galloyl-β-D-glucopyranoside (β-glucogallin, ester-linked gallic acid and glucose) by a UDP-glucosyltransferase (UGT) activity. We cloned and biochemically characterized four candidate UGTs from pomegranate (*Punica granatum*), of which only UGT84A23 and UGT84A24 exhibited β-glucogallin forming activities in enzyme assays. Although overexpression and single RNAi knockdown pomegranate hairy root lines of *UGT84A23* or *UGT84A24* did not lead to obvious alterations in punicalagin (the prevalent HT in pomegranate) accumulation, double knockdown lines of the two UGTs resulted in largely reduced levels of punicalagins and bis-hexahydroxydiphenyl glucose isomers. An unexpected accumulation of galloyl glucosides (ether-linked gallic acid and glucose) was also detected in the double knockdown lines, suggesting that gallic acid was utilized by an unidentified UGT activity for glucoside formation. Transient expression in *Nicotiana benthamiana* leaves and immunogold labeling in roots of pomegranate seedlings collectively indicated cytosolic localization of UGT84A23 and UGT84A24. Overall, functional characterization and localization of UGT84A23 and UGT84A24 open up opportunities for further understanding the regulatory control of HT metabolism in plants and its coordination with other biochemical pathways in the metabolic network.

## Introduction

Tannins are phenolic compounds capable of binding to proteins and interacting with cellulose, lignin, pectin, starch and alkaloids [1]. Tannins can be divided into condensed tannins (aka. proanthocyanidins) and hydrolyzable tannins (HTs), based on their biogenetic origins and structural properties [2]. HTs are further defined as gallotannins or ellagitannins according to the phenolic compound(s) esterified to the hydroxyl groups of the core glucose molecule—gallic acid in gallotannins and hexahydroxydiphenic acid (HHDP) in ellagitannins. Besides defending plants against herbivores [3], HTs also exhibit antioxidant, cancer chemopreventive, cardioprotective, anti-inflammatory, antimicrobial and antiviral activities in humans [4–6].

The first committed reaction of HT biosynthesis entails transfer of the galloyl moiety of 1-*O*-galloyl-β-D-glucopyranoside (β-glucogallin) to a second β-glucogallin molecule [7]. Previous studies in oak (*Quercus robur*) leaves demonstrated that β-glucogallin was produced from conjugation of gallic acid and UDP-glucose by a UDP-glucosyltransferase (UGT) activity [8, 9]. The UGT activity was specific for UDP-glucose as the sugar donor, but recognized various benzoic acid (BA) and cinnamic acid derivatives as glucose acceptors. In addition, the glucosyl transfer reactions yielded glucose esters (i.e. ester-linked glucose and phenolic acids; e.g. β-glucogallin), not phenolic glucosides (i.e. ether-linked glucose and phenolic acids) [8–10]. Genes encoding β-glucogallin forming UGTs have been cloned and biochemically characterized from grape (*Vitis vinifera*; *Vv*gGT1-3), tea (*Camellia sinensis*; UGT84A22) and oak (*Q*. *robur*; UGT84A13) and were proposed to be involved in the biosynthesis of galloylated proanthocyanidins and HTs, respectively [11–13]. Interestingly, the recombinant grape, tea and oak UGTs also synthesized glucose esters from a variety of BA and cinnamic acid derivatives [11–13]. However, the grape, oak and tea UGT activities towards β-glucogallin formation have thus far not been investigated through manipulation of gene expression in tissue cultures or the native plant systems.

**Fig 1:**
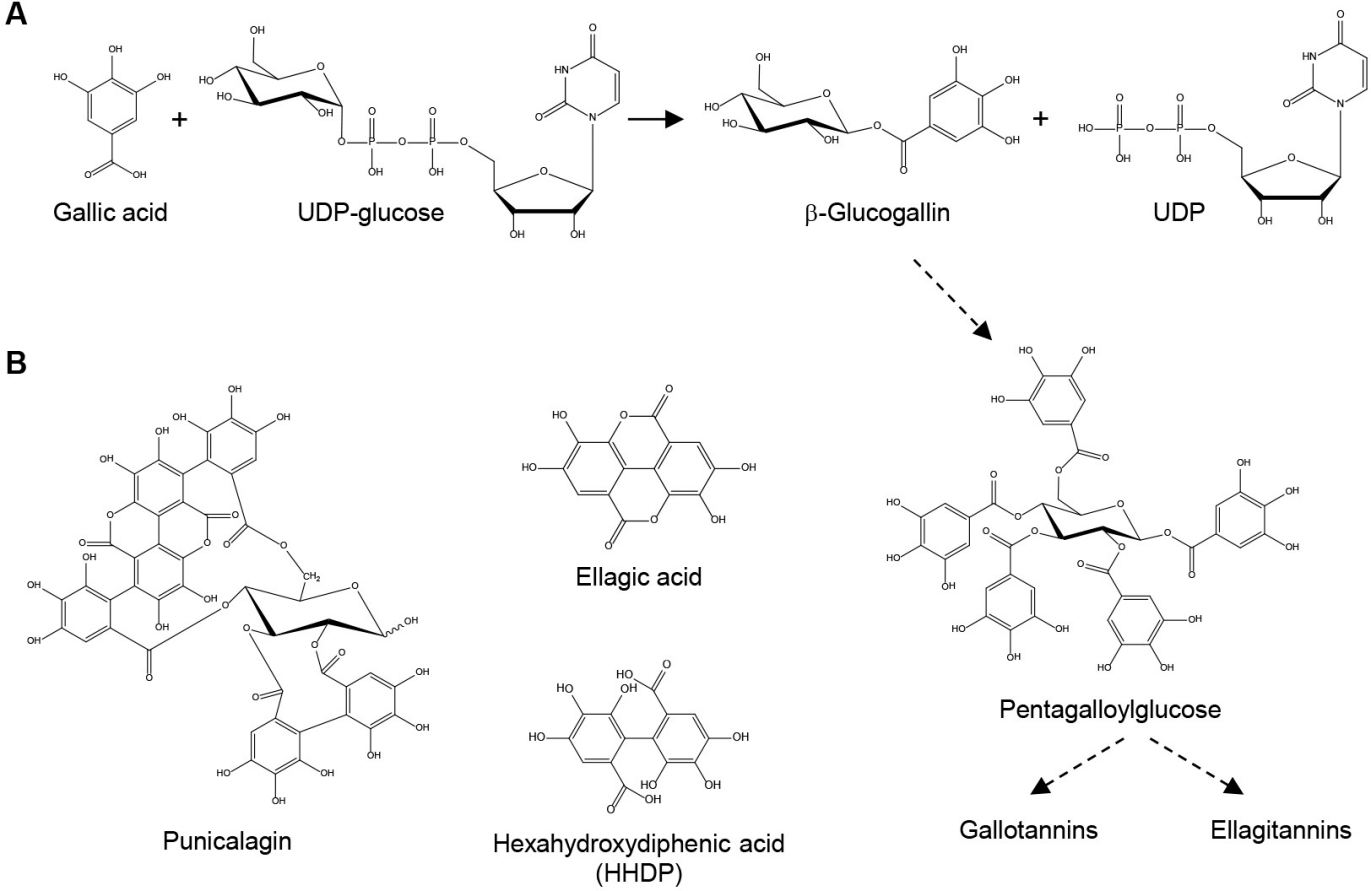
Biosynthesis of hydrolyzable tannins. (A) An ester bond is formed between gallic acid and glucose by a UDP-glucosyltransferase (UGT) activity to produce β-glucogallin. Multiple acyltransferases convert β-glucogallin to pentagalloylglucose, which is further transformed into gallotannins or ellagitannins. Dotted arrows denote multiple enzymatic steps. (B) Chemical structures of punicalagin (α and β isomers), ellagic acid and hexahydroxydiphenic acid (HHDP) are illustrated.

To investigate the localization of HT biosynthesis, an immunohistochemical study was carried out by analyzing the location of pentagalloylglucose (PGG), an intermediate in the HT biosynthetic pathway, and the acyltransferase protein (β-glucogallin:1,2,3,6-tetragalloylglucose 4-*O*-galloyltransferase) that catalyzes its formation [14, 15] (Fig 1). The immunohistochemical results indicated that chloroplast, cell wall and intercellular space were sites for biosynthesis and deposition of HTs. However, it was not clear whether the polyclonal antibody raised against the PGG forming acyltransferase could cross-react with other acyltransferases sharing conserved amino acid sequences in oak leaves. As such, the subcellular sites for HT biosynthesis, especially those of earlier pathway steps (e.g. the UGT catalyzed reaction), remain to be further elucidated.

Pomegranate (*Punica granatum*) is a rich source of HTs, with punicalagin isomers being the dominant HTs present in most tissues [4, 16] (Fig 1). To systematically investigate the HT biosynthetic pathway, we previously established genomic and genetic platforms in pomegranate to assist in candidate gene discovery and characterization in a plant tissue culture system [17, 18]. We here report the cloning, functional characterization and localization of two UGTs, UGT84A23 and UGT84A24, which contribute to β-glucogallin formation in pomegranate.

## Materials and Methods

### Chemicals

Chemicals of analytical grade were purchased from the following vendors: gallic acid, VWR, Radnor, PA; UDP-glucose, Sigma Aldrich, St. Louis, MO; punicalagin, ChromaDex, Irvine, CA; β-glucogallin, CarboSynth, Berkshire, UK; gallic acid-3-*O*-β-D-glucopyranoside and gallic acid-4-*O*-β-D-glucopyranoside, LC Scientific, Ontario, Canada.

### Phylogenetic analysis

Protein sequence alignment was performed using Multiple Sequence Comparison by Log-Expectation (MUSCLE) [19] and was utilized to generate a Neighbor Joining (NJ) phylogenetic tree in MEGA5 [20]. The GenBank (non-Arabidopsis sequences) or AGI (Arabidopsis sequences) accession numbers of the UGTs are: *Db*UGT71F2 (AAL57240), *Mt*UGT71G1 (AAW56092), *At*UGT72B1 (AT4G01070), *Db*UGT73A5 (CAB56231), *Gt*UGT73A14 (BAC54092), *Ac*UGT73G1 (AAP88406), *At*UGT74F1 (AT2G43840), *At*UGT74F2 (AT2G43820), *At*UGT75B1 (AT1G05560), *At*UGT75B2 (AT1G05530), *At*UGT75D1 (AT4G15550), *At*UGT76C2 (AT5G05860), *Sr*UGT76G1 (AAR06912), *At*UGT78D1 (AT1G30530), *Mt*UGT78G1 (ABI94025), *At*UGT82A1 (AT3G22250), *At*UGT83A1 (AT3G02100), *At*UGT84A1 (AT4G15480), *At*UGT84A2 (AT3G21560), *At*UGT84A3 (AT4G15490), *Qr*UGT84A13 (KF527849), *Pg*UGT73AL1 (KT159806), *Cs*UGT84A22 (ALO19890), *Pg*UGT84A23 (KT159805), *Pg*UGT84A24 (KT159807), *Pg*UGT85K15 (KT159804), *At*UGT84B1 (AT2G23260), *Sb*UGT85B1 (AAF17077), *Mt*UGT85H2 (ABI94024), *At*UGT86A1 (AT2G36970), *At*UGT87A1 (AT2G30150), *At*UGT89B1 (AT1G73880), *At*UGT89C1 (AT1G06000), *At*UGT90A2 (AT1G10400), *At*UGT91C1 (AT5G49690), *At*UGT92A1 (AT5G12890), *Pv*UGT93A1 (AAD51778), *Pl*UGT93A2 (AAD04166), *Zm*UGT93B1 (AAK53551), *Bp*UGT94B1 (BAD77944), *Fa*GT2 (AAU09443), *Vl*RSGT (ABH03018), *Vv*GT1 (AAB81683), *Vv*gGT1 (AEW31187), *Vv*gGT2 (AEW31188), *Vv*gGT3 (AEW31189). *Ac*, *Allium cepa*; *At*, *Arabidopsis thaliana*; *Bp*, *Bellis perennis*; *Cs*, *Camellia sinensis*; *Db*, *Dorotheanthus bellidiformis*; *Fa*, *Fragaria × ananassa; Gt*, *Gentiana triflora*; *Mt*, *Medicago truncatula*; *Pg*, *P*. *granatum*; *Pl*, *Phaseolus lunatus*; *Pv*, *Phaseolus vulgaris*; *Qr*, *Quercus robur*; *Sb*, *Sorghum bicolor*; *Sr*, *Stevia rebaudiana*; *Vl*, *Vitis labrusca*; *Vv*, *V*. *vinifera*; *Zm*, *Zea mays*.

### UGT enzyme assays and kinetic analysis

The coding sequences (CDS) of the candidate *PgUGTs* were obtained using Rapid Amplification of cDNA Ends (RACE) (SMARTer RACE cDNA Amplification Kit, Clontech, Mountain View, CA) and inverse PCR reactions (S1 Table), and were cloned into pHis8 or its parent vector pET28a [21]. The plasmid constructs were verified by DNA sequencing and transformed into *E*. *coli* Rosetta 2 (DE3) pLysS cells. Bacterial cell cultures were grown at 37°C to an OD_600_ of 0.6–0.8 and induced with 0.5 mM isopropyl β-D-1-thiogalactopyranoside (IPTG). The cells continued to grow overnight at 16°C and were harvested by centrifugation at 3,000 x g at 4°C for 20 min. His-tagged proteins were purified using the MagneHis protein purification kit (Promega, Madison, WI) and quantified by the Bradford assay (Pierce Coomassie Plus assay kit, Thermo Scientific, Rockford, IL). Purified recombinant proteins were used immediately for *in vitro* assays or frozen at -80°C in 10% glycerol.

The initial UGT enzyme assays (100 μL) included 50 mM Tris-HCl, pH 7.0, 1 mM each of gallic acid and UDP-glucose, 14 mM 2-mercaptoethanol and 10 μg recombinant proteins. The reaction proceeded for 1 h at 30°C and was stopped by adding 10 μL of 100% (w/v) trichloroacetic acid (TCA) and 100 μL of 50% methanol. The mixture was centrifuged at 17,000 x g for 10 min and 10 μL of the supernatant was injected on HPLC. For alkaline hydrolysis of the enzyme assay products, 100 μL of 0.2 N NaOH was added to 100 μL of the UGT assay mixture (after 1 h incubation at 30°C) or the galloyl glucose conjugate standards, incubated for 5 min or 1 h at room temperature, neutralized with 3 M sodium acetate buffer (pH 5.2) and analyzed by HPLC.

The effect of freezing and thawing on UGT activities was determined by comparing freshly purified UGT proteins and those previously frozen in glycerol once at -80°C after purification. The optimal temperature for UGT enzyme assays was evaluated by incubating the reaction mixtures at 0°C and 20°C—55°C with 5°C increments, at pH 7. The effect of pH on UGT activities towards gallic acid was determined at pH 3.5–8 with 0.5 pH increments, at 30^°^C. The specific activity (nkat/mg) of the UGT proteins was calculated as nmol substrate converted/s (nkat) by 1 mg of purified protein. Three independent reactions were performed for each enzyme assay. In addition to gallic acid, selected phenolic acids and phenylpropanoids were also tested as substrates for glucosyl transfer reactions, including BA, 2-hydroxybenzoic acid (2-HBA), 3-HBA, 4-HBA, 2,3-dihydroxybenzoic acid (2,3-DHBA), 2,4-DHBA, 2,5-DHBA, 2,6-DHBA, 3,4-DHBA, cinnamic acid, coumaric acid, caffeic acid, ferulic acid, sinapic acid, catechol, chlorogenic acid, resveratrol, genistein, naringenin, quercetin, apigenin, luteolin, catechin, epicatechin, pelargonidin, cyanidin and delphinidin.

For measuring steady-state kinetics of UGT84A23 and UGT84A24, the enzyme assay mixture (100 μL) contained 50 mM MES, pH 5.0, 1 mM UDP-glucose, 0.1 mM– 1.2 mM phenolic acid substrate, 14 mM 2-mercaptoethanol and 4 μg purified recombinant proteins. UGT84A23 and UGT84A24 enzyme activities were linear at 30°C for at least 60 min. Therefore, triplicate reactions were carried out at 30°C for 30 min, terminated and analyzed on HPLC as described above. The kinetic parameters were calculated using Hyper32 based on the Linewaver-Burk plot (http://homepage.ntlworld.com/john.easterby/hyper32.html).

### Hairy root transformation

To overexpress *UGT84A23* or *UGT84A24* in pomegranate hairy roots, the CDS of the two *PgUGTs* were cloned into pK7WG2D under the control of a *35S* promoter [22] (S1 Table). For down-regulation of *UGT84A23* or *UGT84A24* expression, fragments of 300–450 bp from the CDS or the 3’ untranslated region (UTR) of each gene were incorporated in the hairpin RNAi vector pHG8-YFP [23]. In addition, two chimeras of *UGT84A23* and *UGT84A24* CDS or 3’ UTR sequences were generated using overlap extension PCR and cloned into pHG8-YFP. *Agrobacterium rhizogenes* MSU440 cells were transformed with the empty vectors, the overexpression or RNAi knockdown constructs by electroporation, and used for transformation of pomegranate radicles [18]. One transgenic root per explant was maintained to ensure that the tissue harvested and analyzed derived from an independent transformation event.

### HPLC, MS and MS/MS analyses

Gradients between two solvents, water containing 0.1% formic acid (A) and 100% acetonitrile (B), and a flow rate of 1 mL min^-1^ were used in HPLC analyses. For analyzing reaction products using gallic acid as substrate, the HPLC gradient was 0–3 min, 5% B, 3–14 min, 5–15% B, 14–15 min, 15–5% B. A modified gradient was used to separate reaction products of various phenolic acid and flavonoid substrates: 0–3 min, 5% B, 3–17.5 min, 5–50% B, 17.5–18 min, 50–5% B. Identification of glucosylated products were based on retention time and UV spectrum, by comparison to either the authentic standards or the aglycones, where the conjugates show similar UV spectra to the aglycones but with altered retention time, as well as MS and MS/MS analyses. For metabolite analysis of pomegranate hairy roots, total phenolic compounds were extracted from 100 mg of ground pomegranate tissues with 300 μL of 40% methanol. The mixture was incubated in an ultrasonic water bath for 20 min at 30°C and then centrifuged twice for 10 min each at 17,000 x g. Ten μL of the supernatant was analyzed at 0–3 min, 5% B, 3–24 min, 5–25% B, 24–29 min, 25–35% B, 29–30 min, 35–5% B.

MS and MS/MS analyses were performed using the Thermo Electron LTQ-Orbitrap Hybrid mass spectrometer (Thermo Scientific). Product peaks from the UGT assays or peaks of interest in wild type and transgenic hairy roots were collected by HPLC, concentrated under vacuum and used for injection onto the mass spectrometer. An isocratic flow of an equal volume of solvents A and B was maintained at 0.2 mL min^-1^ for MS analysis and syringe pump injections were made at 10 μL min^-1^ for MS/MS analysis. The mass spectra were acquired by electrospray ionization (ESI) in the negative mode and a mass range of *m/z* 120–1,000 Da (MS) or 90–1,000 Da (MS/MS) was used for the mass spectral analysis. The normalized collision energy for MS/MS analysis was 18–20%.

### Expression analysis

Total RNA was extracted from hairy roots using a CTAB-based method as previously described [24]. The RNA quantity (OD_260_) and quality (OD ratios at 260/280 nm and 260/230 nm) were determined on a NanoDrop^®^ spectrophotometer and by visual inspection of RNA integrity separated on a 1% agarose gel. Total RNA was treated with DNase I (Fermentas, Glen Burnie, MD) and 1 μg total RNA was reverse transcribed into cDNA with an oligo (dT)_20_ primer using Superscript^®^ III (Invitrogen).

qPCR primers for *UGT84A23*, *UGT84A24* and the reference genes were designed using Primer Express 2.0 (S2 Table). qPCR reactions were performed with iTaq^TM^ Universal SYBR^®^ Green supermix (BioRad, Hercules, CA), 0.8 μL cDNA and 200 nM each primer on the ABI Prism^®^ 7300 Real-Time qPCR System (Applied Biosystems, Foster City, CA). Cycling parameters were as follows: 1 cycle of 30 sec at 95°C, followed by 40 cycles of 15 sec at 95°C and 1 min at 60°C. The primer amplification efficiency for the UGTs and reference genes was in the range of 93% to 106%. Dissociation curves were run for all primer pairs to verify the presence of a single amplified peak. The qPCR products were also cloned and sequenced to confirm amplifications of the target genes. Expression of pomegranate homologs of *ubiquitin C4* (*UBC4*), *phosphoglycerate kinase* (*PGK*), *glyceraldehyde 3-phosphate dehydrogenase* (*GAPD*) and *TATA-binding protein* (*TBP*) was determined and assessed for relative stability across multiple non-transgenic and transgenic hairy root lines. *UBC4* and *PGK* were the most stable reference genes with C_t_ deviations less than 0.5 across all samples analyzed. The relative expression of *UGT84A23* and *UGT84A24* across different hairy root lines was therefore determined using the comparative C_t_ (ΔΔC_t_) method with the geometric mean of *UBC4* and *PGK* for normalization and the vector transformed hairy root control as calibrator [25]. Semi-quantitative reverse transcription (RT)-PCR analysis of *UGT84A24* expression was performed as previously described [18].

### Subcellular localization

The CDS of *UGT84A23* or *UGT84A24* were cloned into the pGWB5 and pGWB6 vectors, with or without stop codons, for expression as N- or C-terminal green fluorescent protein (GFP) fusions [26]. The plasmid constructs were electroporated into *Agrobacterium tumefaciens* GV3101 cells; agroinfiltration of 1-mon-old pomegranate or *N*. *benthamiana* leaves was conducted as previously described [27]. The infiltrated leaves were examined under a Zeiss LSM 710 AxioObserver confocal microscope equipped with a LD C-Apochromat 40x/1.1 W Korr M27 objective (Zeiss, Jena, Germany). Wavelengths used for excitation were 488 nm for green fluorescence, 561 nm for red fluorescence and 440 nm for chlorophyll autofluorescence. Detection wavelengths were 497–531 nm for green fluorescence, 610–640 nm for red fluorescence and 660–680 nm for chlorophyll autofluorescence. Images were processed for presentation using the Zen 2012 software.

A polyclonal antibody against purified recombinant UGT84A24 proteins was produced in a rabbit (YenZyme, South San Francisco, CA). Pre-immune serum, test and final bleeds were utilized for antigen-specific ELISAs to verify antibody recognition of UGT84A24. The antibody was purified from anti-serum using the Pierce MicroLink^®^ peptide coupling kit (Thermo Scientific) with UltraLink^®^ iodoacetyl gel bound with purified recombinant UGT84A24 proteins. Transverse and longitudinal sections from roots of 1-mon-old pomegranate seedlings were fixed with 4% paraformaldehyde and 0.5% glutaraldehyde in 0.1 M sodium phosphate buffer using a microwave-assisted method [28]. The fixed samples were treated with 0.1% tannic acid, dehydrated with ethanol, and subsequently infiltrated with and embedded in LR White resins (Sigma). Ultrathin sections (80–90 nm) were cut using a diamond knife (Diatome, Biel, Switzerland) on a Ultracut UCT ultramicrotome (Leica microsystems, Bannockburn, IL), and mounted on formvar coated gold grids (Electron Microscopy Sciences, Hatfield, PA). The grids were incubated with 1.5% goat serum in phosphate buffered saline (PBS) for 30 min at room temperature before incubation with the primary antibody solution [a 1:5 (v/v) dilution of anti-UGT84A24 mixed with either 1.5% goat serum or 2 μg μL^-1^ of the recombinant UGT84A23 and UGT84A24 proteins] overnight at 4°C. A without-primary-antibody control (i.e. incubation with 1.5% goat serum only) was processed in parallel. The grids were rinsed with PBS and then incubated with 10 nm gold-labeled anti-rabbit secondary antibody in PBS [British Bio-Cell International, Cardiff, UK; 1:50 (v/v) dilution] at room temperature for 1 h followed by rinsing with PBS and water. All grids were stained for 30 min with 4% uranyl acetate in 70% ethanol and for 2 min with lead citrate before observation on a Phillips CM120 Biotwin at 80 kV. Electron micrographs were taken with a Gatan MegaScan digital camera (model 794/20). The number of immunogold particles in each of the 7 defined compartments per 9 μm^2^ image area was counted using ImageJ. Between-group comparisons (anti-UGT84A24 vs. the antigen-saturated control) were carried out using one-way ANOVA and χ^2^ analysis as described in [29].

### Statistical analysis

Integrated peak values were exported from ChemStation (Agilent, Santa Clara, CA). Isomeric peaks of punicalagins were combined for statistical analysis. The peak values were assessed for normal distribution by the Shapiro-Wilk test for the vector transformed control, overexpression or knockdown hairy root lines. Log-transformed peak values were also subjected to the Shapiro-Wilk test and resulted in a slightly improved test result, with *W* > 0.95 (indicating normally distributed data) for all peaks analyzed. The log-transformed peak values were then examined for difference in group means by one-way ANOVA. The Shapiro-Wilk and ANOVA tests were performed using SAS (Cary, NC).

## Results

### UGT84A23 and UGT84A24 converted gallic acid and UDP-glucose to β-glucogallin

**Fig 2:**
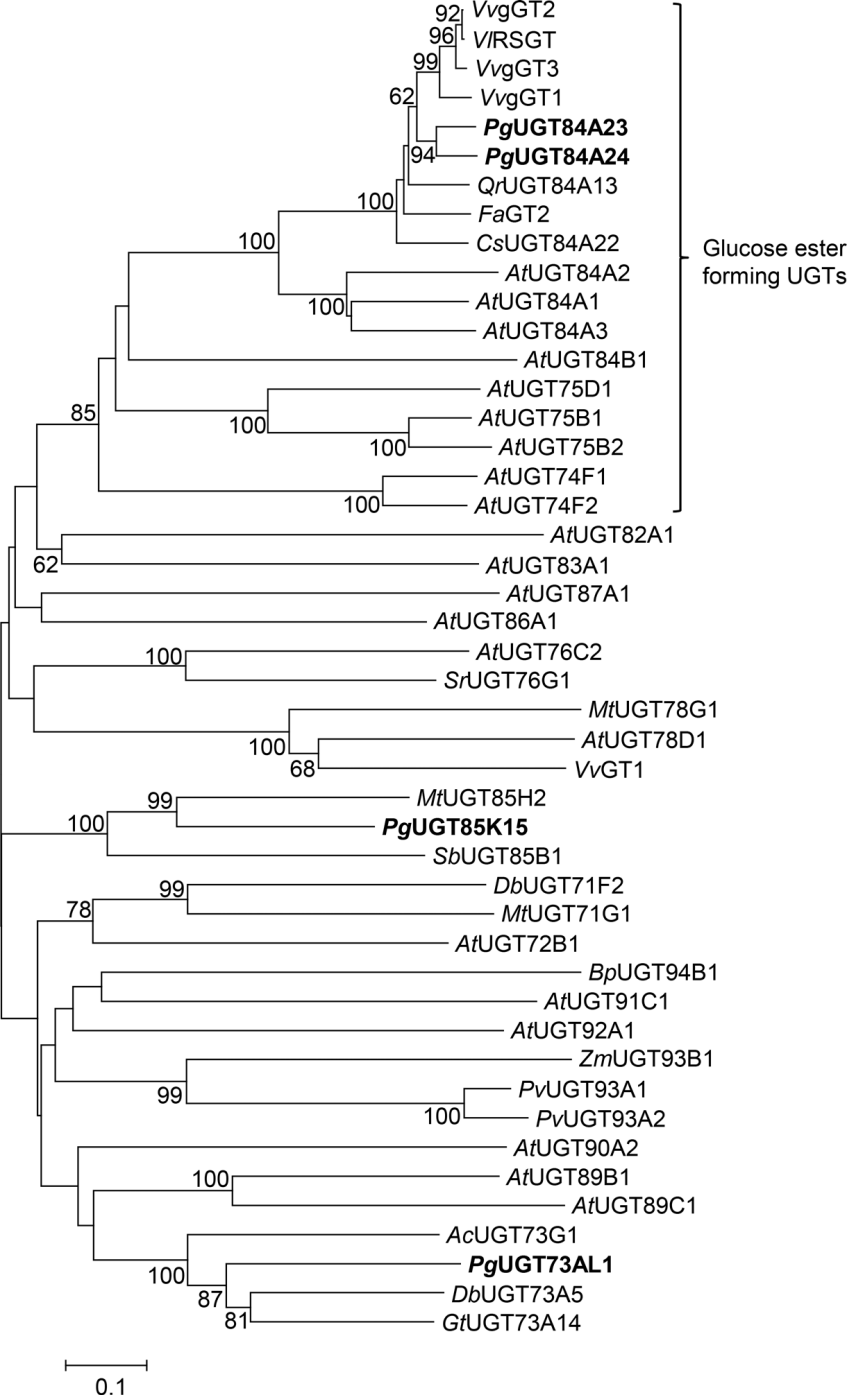
Pomegranate UGT84A23 and UGT84A24 cluster with glucose ester forming UGTs. A neighbor-joining (NJ) tree was constructed using PgUGTs with selected plant family 1 UGTs that represent fifteen phylogenetic groups [30] and glucose ester forming UGTs that recognize phenolic acids as substrates. Bootstrap values (1,000 iterations) greater than 60% are shown next to the branches. UGT84A23, UGT84A24, UGT73AL1 and UGT85K15 are highlighted in bold text.

We previously utilized a transcriptome of pomegranate fruit peel, the tissue that exhibits the highest accumulation of punicalagins, to identify 26 putative PgUGTs [18]. These PgUGTs clustered with 7 phylogenetic groups (B, D, E, F, G, L and M) when compared with plant family 1 UGTs [17, 30]. Since transcriptional control has been suggested to be the primary mode of regulation for specialized metabolite accumulation [31], *UGT85K15* (formerly *PgUGT1*), *UGT84A23* (formerly *PgUGT13*) and *UGT73AL1* (formerly *PgUGT16*) were proposed to be involved in HT biosynthesis as their transcript accumulation either correlated with punicalagin levels in different pomegranate tissues or was very high in fruit peel (Ono et al, 2012). UGT84A23 was further corroborated as a strong candidate along with its homolog UGT84A24 (formerly PgUGT17, sharing 85% protein sequence identity with UGT84A23) since both clustered with glucose ester-forming UGTs from other plant species (Fig 2). Therefore, UGT85K15, UGT73AL1, UGT84A23 and UGT84A24 were considered strong candidates for β-glucogallin biosynthesis in pomegranate.

**Fig 3:**
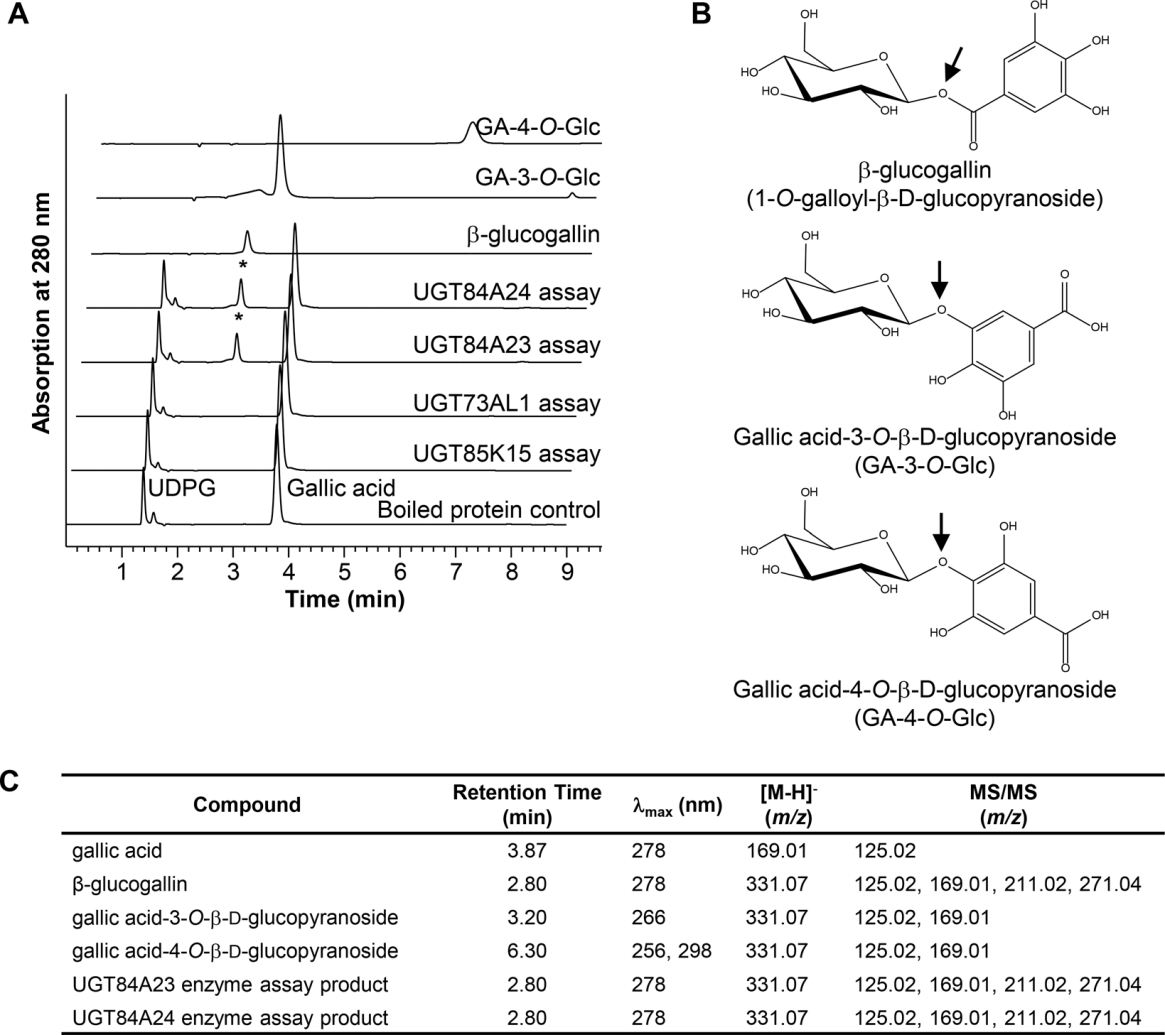
Pomegranate UGT84A23 and UGT84A24 convert UDP-glucose (UDPG) and gallic acid to β-glucogallin. (A) HPLC elution profiles of the enzyme assay products (indicated with asterisks) and the authentic standards at 280 nm are shown at a 1% offset retention time and 50% offset absorbance. (B) Chemical structures of galloyl glucose conjugates. The ester (β-glucogallin) or ether (GA-3-*O*-Glc and GA-4-*O*-Glc) linkages between gallic acid and glucose are indicated by arrows. (C) Pomegranate UGT84A23 and UGT84A24 enzyme assay products were identified via HPLC, MS and MS/MS analyses.

The four candidate UGTs were expressed as His-tagged proteins in *E*. *coli* and purified for *in vitro* enzyme assays (Fig 3, S1 Fig). When incubated with gallic acid and UDP-glucose, UGT84A23 and UGT84A24, but not UGT85K15 or UGT73AL1, exhibited activities towards the substrates and formed a product (Fig 3A). Crude protein extracts of *E*. *coli* cells expressing UGT85K15 or UGT73AL1 were also used in the enzyme assays and did not show any activity towards gallic acid and UDP-glucose (data not shown). Mass spectrometry (MS) analysis of the UGT84A23 and UGT84A24 enzyme assay products showed an [M-H]^-^ at *m/z* 331.07, which is consistent with a galloyl glucose conjugate (Fig 3C, S2 Fig). When compared to standards of three galloyl glucose conjugates, the retention times of the enzyme reaction products in HPLC analysis matched to that of β-glucogallin (2.80 min), but not gallic acid-3-*O*-β-D-glucopyranoside (GA-3-*O*-Glc, 3.20 min) or gallic acid-4-*O*-β-D-glucopyranoside (GA-4-*O*-Glc, 6.30 min) (Fig 3C). The UGT84A23 and UGT84A24 enzyme assay products ([M-H]^-^ at *m/z* 331.07) were also subjected to MS/MS analysis to further verify the linkage connecting gallic acid and glucose. For both UGT84A23 and UGT84A24 enzyme assay products, daughter ion spectra (*m/z* 125.02, 169.01, 211.02 and 271.04) agreed with that of β-glucogallin, but not of the galloyl glucose ethers (Fig 3C, S2 Fig). Overall, the HPLC, MS and MS/MS data collectively indicated that UGT84A23 and UGT84A24 produced the galloyl glucose ester, β-glucogallin, from gallic acid and UDP-glucose in *in vitro* enzyme assays. It is worth noting that UGT84A23 and UGT84A24 enzyme assay products were also treated with NaOH to verify the galloyl glucose linkage as glucose esters are generally less stable than glucose ethers under alkaline conditions. However, the alkaline hydrolysis results were not clear since the enzyme assay products and the three galloyl glucose conjugate standards were mostly degraded after only a 5-min incubation of 0.2 N NaOH (S3 Fig).

To determine whether UGT84A23 and UGT84A24 activities are retained when frozen in glycerol, enzyme assays were carried out using thawed UGT proteins previously stored in glycerol at -80°C (freeze-thawed). Both freeze-thawed and freshly prepared UGT proteins possessed comparable enzyme activities (S4 Fig). UGT84A23 and UGT84A24 showed little variation in their specific activities when incubated at 20°C—55°C (S5 Fig). Interestingly, the two UGTs were still active when the enzyme reactions were conducted at 0°C (S5 Fig). While the highest specific activity of UGT84A23 with gallic acid and UDP-glucose was achieved at pH 5, the greatest specific activity for UGT84A24 was obtained at pH 4.5 (S6 Fig).

**Fig 4:**
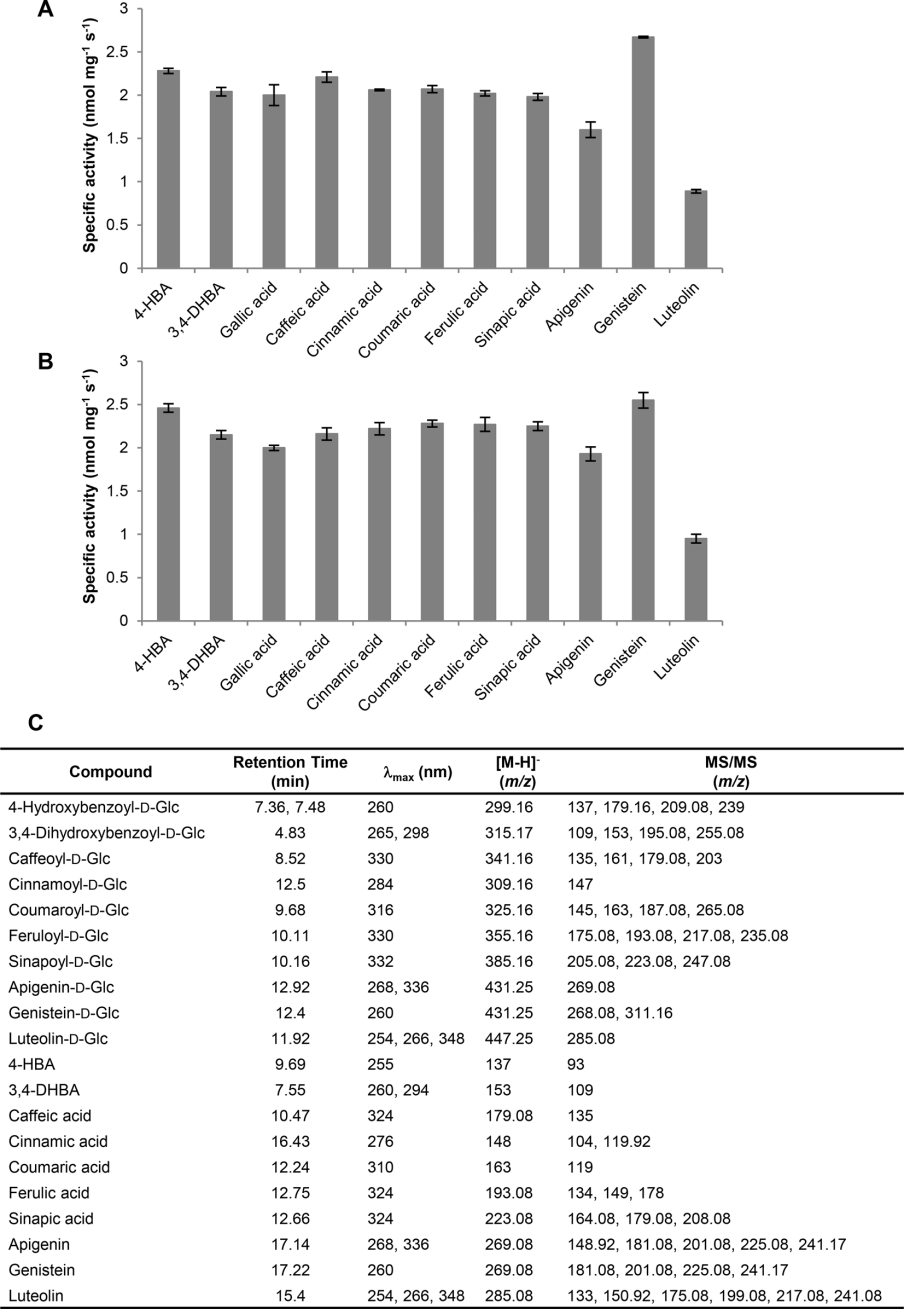
Pomegranate UGT84A23 and UGT84A24 are active towards a range of phenolic substrates. Specific activities of recombinant UGT84A23 (A) and UGT84A24 (B) at pH 5 (phenolic acids) and pH 8 (flavonoids) are shown. C. HPLC retention times, maximum absorbance (λ_max_), MS and MS/MS fragmentation patterns of the phenolic substrates and their glucose (Glc) conjugates formed by UGT84A23 and UGT84A24. 4-HBA, 4-hydroxybenzoic acid; 3,4-DHBA, 3,4-dihydroxybenzoic acid.

The range of UGT84A23 and UGT84A24 substrate recognition was then examined by using various BA and cinnamic acid derivatives as well as selected phenylpropanoids as sugar acceptors in enzyme assays. UGT84A23 and UGT84A24 exhibited significant and comparable activities towards 4-HBA, 3,4-DHBA, cinnamic acid and its derivatives, genistein, apigenin and luteolin (Fig 4). MS and MS/MS fragmentation patterns of the reaction products using phenolic acids as substrates resembled the glucose esters formed by *Vv*gGT1-3 and *Fa*GT2 (a strawberry UGT that produces glucose esters from various phenolic acid substrates) [11, 32] (Fig 4C). On the other hand, flavonoid glucosides were generated when genistein, apigenin and luteolin served as sugar acceptors (Fig 4C). UGT84A23 and UGT84A24 also showed low activities on BA, 2-HBA, 3-HBA, 2,4-DHBA, naringenin and quercetin (S7 and S8 Figs). However, they did not function towards catechol, resveratrol, chlorogenic acid, catechin and epicatechin (building blocks of proanthocyanidins) or the three anthocyanidins (cyanidin, delphinidin and pelargonidin) that were tested (data not shown).

Steady-state kinetics of UGT84A23 and UGT84A24 were further investigated on phenolic acid substrates that showed substantial specific activities (≥ 1 nmol mg^-1^ s^-1^) (Fig 4, Table 1). While UGT84A23 has the highest affinities to gallic acid (*K*m = 0.89 ± 0.07 mM) and sinapic acid (*K*m = 0.86 ± 0.05 mM), UGT84A24 is most affiliated with sinapic acid (*K*m = 0.78 ± 0.02 mM) and has relatively high affinity with gallic acid (*K*m = 0.98 ± 0.01 mM). UGT84A23 and UGT84A24 generally share similar Michaelis-Menten kinetic parameters (*K*m, *V*max, *k*cat and *k*cat/*K*m) towards different substrates (Table 1). One notable exception is 3,4-DHBA to which the two UGTs showed 2–4 fold lower affinity, but higher turnover (*k*cat = 0.76 ± 0.06 s^-1^ and *k*cat = 1.35 ± 0.19 s^-1^, respectively), than the other substrates. As a result of the high *K*m values, the *k*cat/*K*m for 3,4-DHBA was the lowest among all the substrates examined for UGT84A23 and UGT84A24 (Table 1).

**Table 1:** Kinetic parameters of pomegranate UGT84A23 and UGT84A24 towards selected phenolic acid substrates.

	UGT84A23				UGT84A24			
Substrate	*K*_m_ (mM)	*V*_max_ (μM s^-1^)	*k*_cat_ (s^-1^)	*k*_cat_/*K*_m_ (mM^-1^ s^-1^)	*K*_m_ (mM)	*V*_max_ (μM s^-1^)	*k*_cat_ (s^-1^)	*k*_cat_/*K*_m_ (mM^-1^ s^-1^)
Gallic acid	0.89 ± 0.07	0.37 ± 0.02	0.52 ± 0.03	0.58	0.98 ± 0.01	0.39 ± 0.01	0.55 ± 0.01	0.56
4-HBA	1.19 ± 0.03	0.43 ± 0.02	0.61 ± 0.03	0.51	1.17 ± 0.03	0.43 ± 0.01	0.6 ± 0.01	0.51
3,4-DHBA	2.46 ± 0.26	0.54 ± 0.04	0.76 ± 0.06	0.31	4.44 ± 0.73	0.96 ± 0.14	1.35 ± 0.19	0.3
Caffeic acid	1.06 ± 0.11	0.45 ± 0.04	0.64 ± 0.05	0.6	1.77 ± 0.17	0.63 ± 0.06	0.89 ± 0.08	0.5
Cinnamic acid	1.12 ± 0.08	0.4 ± 0.02	0.56 ± 0.02	0.5	0.86 ± 0.09	0.32 ± 0.03	0.45 ± 0.04	0.52
Coumaric acid	0.94 ± 0.13	0.37 ± 0.05	0.52 ± 0.07	0.55	1.06 ± 0.15	0.31 ± 0.05	0.44 ± 0.08	0.42
Ferulic acid	1.58 ± 0.01	0.52 ± 0.01	0.72 ± 0.01	0.46	1.32 ± 0.02	0.47 ± 0.01	0.66 ± 0.01	0.5
Sinapic acid	0.86 ± 0.05	0.35 ± 0.02	0.5 ± 0.02	0.58	0.78 ± 0.02	0.31 ± 0.01	0.44 ± 0.01	0.56

4-HBA, 4-hydroxy benzoic acid; 3,4-DHBA, 3,4-dihydroxy benzoic acid.

### UGT84A23 and UGT84A24 demonstrated β-glucogallin forming activities in transgenic pomegranate hairy roots

**Fig 5:**
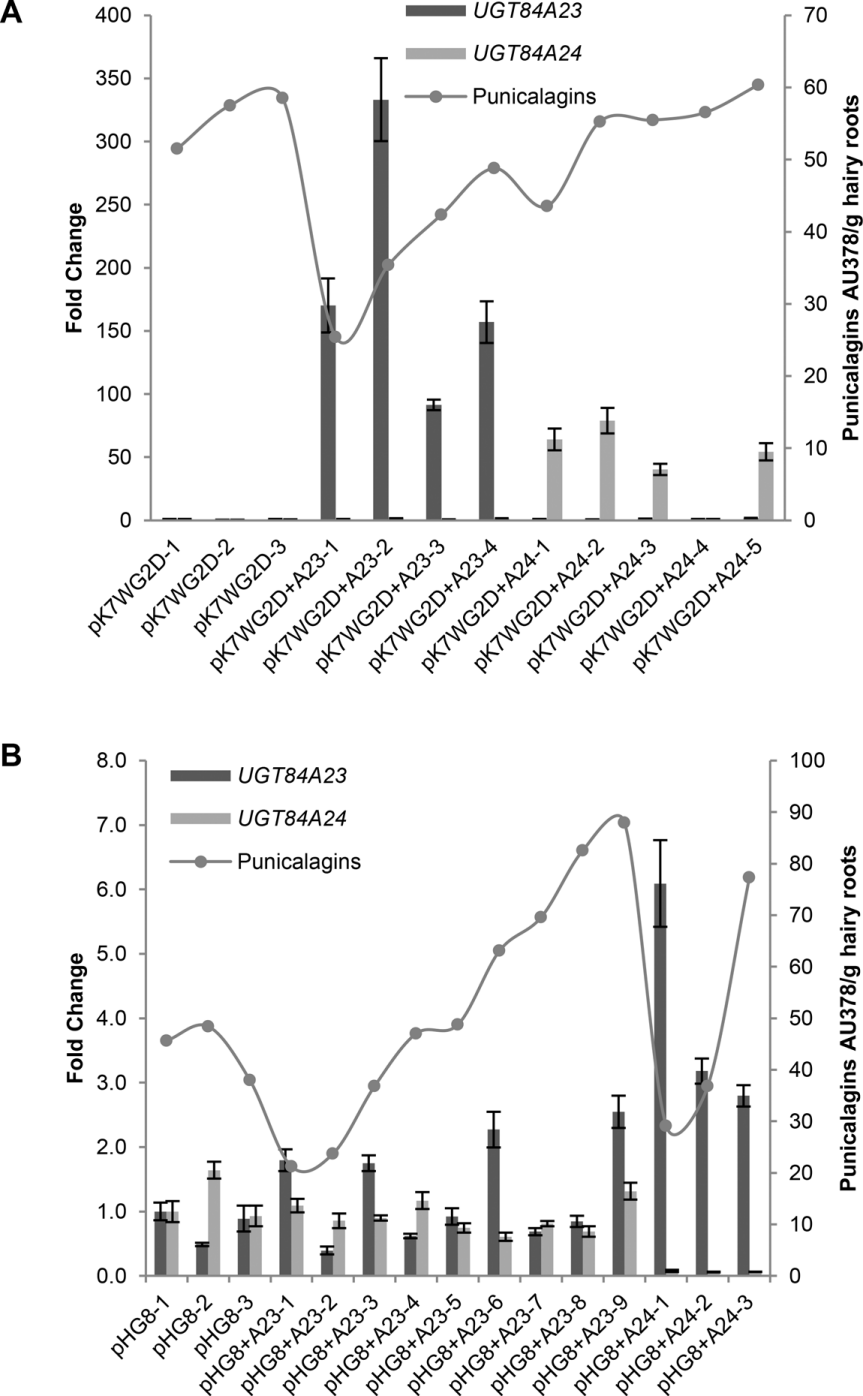
**Gene expression and punicalagin accumulation in overexpression (A) and RNAi knockdown (B) hairy root lines of *UGT84A23* or *UGT84A24***. Changes in gene expression are represented on the primary y-axis as fold change relative to the vector transformed control, pK7WG2D-1 for overexpression lines and pHG8-1 for RNAi knockdown lines. Gene expression data presented are mean ± SD of three technical replicates for each line. Punicalagin accumulation (α and β isomers combined) is represented on the secondary y-axis as peak areas at 378 nm.

**Fig 6:**
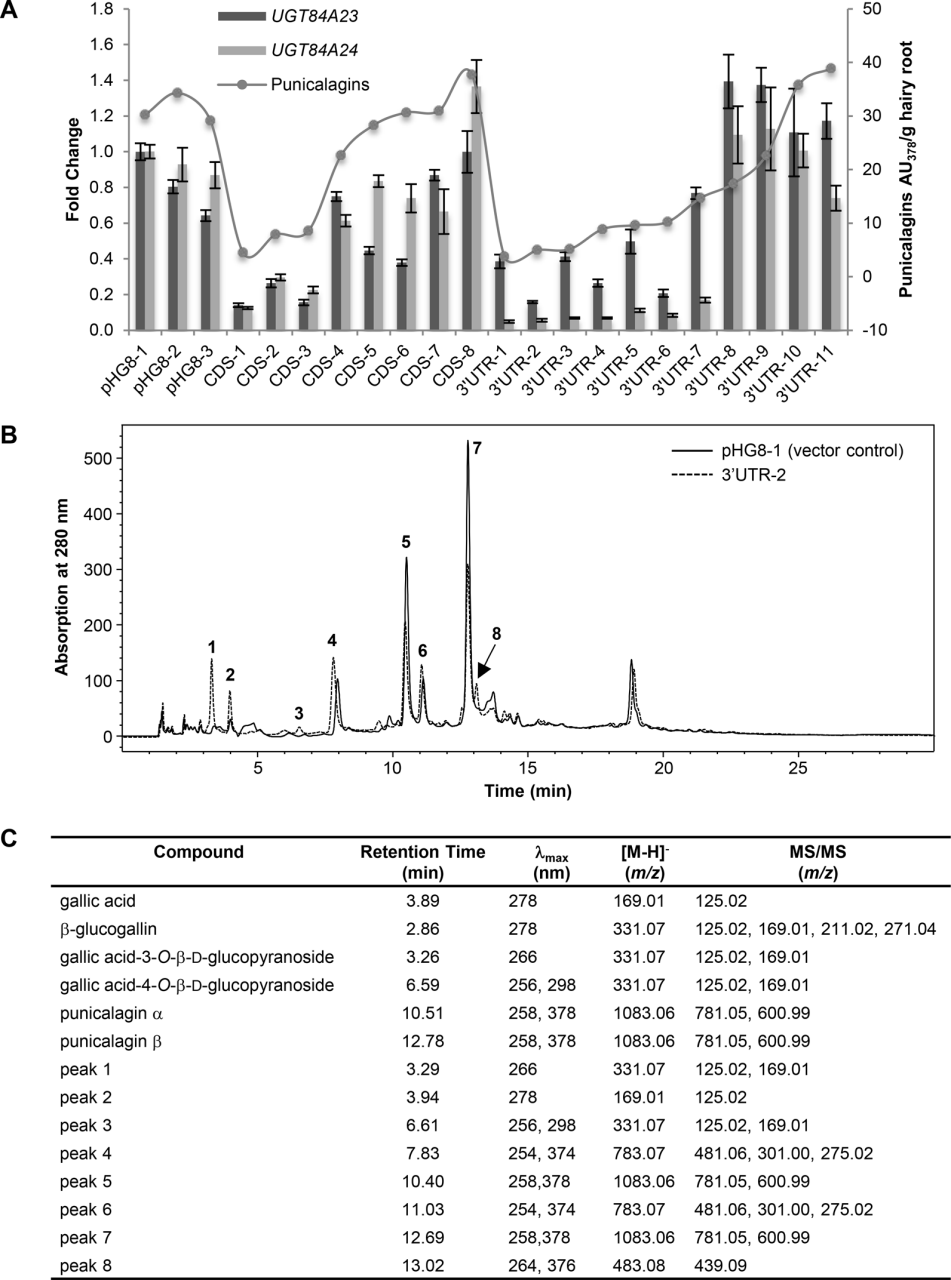
Reduced punicalagin accumulation and increased galloyl glucoside production in *UGT84A23* and *UGT84A24* double RNAi knockdown hairy root lines. (A) Gene expression and punicalagin accumulation in vector control and *UGT84A23* and *UGT84A24* double RNAi knockdown lines. Changes in gene expression are represented on the primary y-axis as fold change relative to the vector control pHG8-1. Gene expression data presented are mean ± SD of three technical replicates for each line. Punicalagin accumulation (α and β isomers combined) is represented on the secondary y-axis as peak areas at 378 nm. The RNAi constructs were derived from a chimera of the coding sequences (CDS) or the 3’ untranslated region (3’ UTR) of *UGT84A23* and *UGT84A24*. (B) Overlay of HPLC chromatograms from a representative vector transformed control (pHG8-1) and a representative double knockdown line of *UGT84A23* and *UGT84A24* (3’ UTR-2). Peaks that are only present in the double knockdown lines and those that show varied accumulation in vector control and double knockdown lines are indicated. (C) MS and MS/MS analyses of peaks indicated in (B). HPLC retention times and λ_max_ of standards and the respective peaks in the 3’ UTR-2 hairy root line are also shown.

To determine whether UGT84A23 and UGT84A24 are involved in HT biosynthesis in pomegranate, expression of the two *UGTs* was manipulated through overexpression or RNAi down-regulation (knockdown) in pomegranate hairy roots (Figs 5 and 6). A total of 3 pK7WG2D vector transformed controls, 6 *UGT84A23* overexpression and 8 *UGT84A24* overexpression hairy root lines were independently generated and analyzed for punicalagin accumulation. A one-way analysis of variation (ANOVA) test showed that there was no statistically significant difference for punicalagin accumulation between the vector transformed controls (51.3 ± 6.3 AU; AU, absorption unit) and the *UGT84A23* (42.6 ± 10.7 AU, *p* = 0.24) or *UGT84A24* (53.0 ± 19.3 AU, *p* = 0.88) overexpression lines. In addition, the general phenolic profiles of the *UGT84A23* and *UGT84A24* overexpression lines were similar to the vector transformed controls (S9 Fig). Three vector transformed controls as well as 4 *UGT84A23* overexpression and 5 *UGT84A24* overexpression lines that showed various levels of punicalagin accumulation were then selected for gene expression analysis (Fig 5A). *UGT84A23* and *UGT84A24* expression was largely enhanced in the respective overexpression lines when compared to the vector transformed controls. However, the increased *UGT84A23* and *UGT84A24* expression did not appear to correlate with punicalagin levels in these hairy root lines (Fig 5A).

To down-regulate *UGT84A23* or *UGT84A24* expression, a total of 9 pHG8 vector transformed controls, 9 *UGT84A23* knockdown and 6 *UGT84A24* knockdown hairy root lines were independently generated and analyzed for punicalagin levels (Fig 5B). Similar to the overexpression lines, the RNAi knockdown lines of *UGT84A23* or *UGT84A24* did not differ significantly in punicalagin accumulation compared to the vector transformed controls (44.8 ± 14.8 AU) based on ANOVA analysis (54.0 ± 25.7 AU, *p* = 0.37 for *UGT84A23* knockdown lines and 41.0 ± 24.0 AU, *p* = 0.71 for *UGT84A24* knockdown lines). In addition, the general phenolic profiles in the RNAi knockdown lines were also similar to the vector transformed controls (S9 Fig). Three vector transformed controls as well as 9 *UGT84A23* knockdown and 3 *UGT84A24* knockdown lines were selected for examination of gene expression (Fig 5B). *UGT84A23* and *UGT84A24* expression levels could not be correlated to punicalagin accumulation in the respective RNAi knockdown lines (Fig 5B).

To determine whether UGT84A23 and UGT84A24 possess overlapping activities and could compensate for each other’s reduced activities in the single RNAi knockdown lines, a total of 22 pHG8 vector transformed controls, 19 CDS double knockdown lines and 22 3’ UTR double knockdown lines were independently generated (Fig 6). There was a significant difference in punicalagin accumulation between the vector transformed controls (34.5 ± 11.4 AU) and the double knockdown lines (24.2 ± 9.2 AU, *p* = 0.0032 for the CDS double knockdown lines and 16.8 ± 10.8 AU, *p* < 0.0001 for the 3’ UTR double knockdown lines) based on ANOVA analysis. Three vector transformed control, 8 CDS double knockdown and 11 3’ UTR double knockdown lines were analyzed for *UGT84A23* and *UGT84A24* gene expression (Fig 6A). *UGT84A23* and *UGT84A24* transcripts were decreased by as much as 7- and 8-fold for the CDS double knockdown lines and 6- and 17-fold for the 3’ UTR double knockdown lines, respectively. Upon examination of *UGT84A23* and *UGT84A24* expression and punicalagin levels, there appeared to be a trend of decreased punicalagin accumulation in hairy root lines with reduced *UGT84A23* and *UGT84A24* expression (Fig 6A).

In addition to strong reduction in punicalagin isomers, changes in several other phenolic metabolites were also observed in the *UGT84A23* and *UGT84A24* double knockdown lines (Fig 6B). Three new compounds appeared at 3.29 min (peak 1), 6.61 min (peak 3) and 13.02 min (peak 8) in the double knockdown lines that were not detectable in the vector transformed hairy roots (Fig 6B). Peaks 1 and 3 are consistent with GA-3-*O*-Glc and GA-4-*O*-Glc according to comparison of the HPLC, MS and MS/MS data for the peaks and standards (Fig 6C). Based on the MS data, peak 8 (*m/z* 483.08) is a digalloyl glucose conjugate [33]. The MS/MS fragmentation pattern further suggested that one of the galloyl groups could be a gallic acid because of the characteristic presence of a *m/z* 439.09 ion derived from losing a carbonyl group from an ether linked gallic acid [34] (Fig 6C). In addition to the three new compounds, significant (marginally significant in the CDS double knockdown lines) increases in compounds eluted at 7.83 min (peak 4) (19.7 ± 7.9 AU for the vector transformed controls; 14.3 ± 8.8 AU, *p* = 0.0468 for the CDS double knockdown lines; 13.2 ± 9.9 AU, *p* = 0.0053 for the 3’ UTR double knockdown lines) and 11.03 min (peak 6) (20.6 ± 6.8 AU for the vector transformed controls; 16.2 ± 8.6 AU, *p* = 0.0743 for the CDS double knockdown lines; 13.2 ± 7.6 AU, *p* = 0.0007 for the 3’ UTR double knockdown lines) were found in the double knockdown lines (Fig 6B). The MS and MS/MS analyses indicated that peaks 4 and 6 are isomers of pedunculagin (i.e. di-HHDP glucose) [33]. Varied accumulation of the compound that eluted at 3.94 min (peak 2) was detected in the double knockdown lines compared to the vector transformed controls (Fig 6B). The elution time of peak 2 matched to that of gallic acid; the identity of peak 2 was further verified by MS and MS/MS analyses (Fig 6C). However, ANOVA analysis indicated that gallic acid accumulation did not differ significantly between the vector transformed controls (4.9 ± 2.8 AU) and the double knockdown lines (3.9 ± 1.7 AU, *p* = 0.1937 for the CDS double knockdown lines and 6.2 ± 3.0 AU, *p* = 0.1044 for the 3’ UTR double knockdown lines).

### UGT84A23 and UGT84A24 are localized in the cytosol

**Fig 7:**
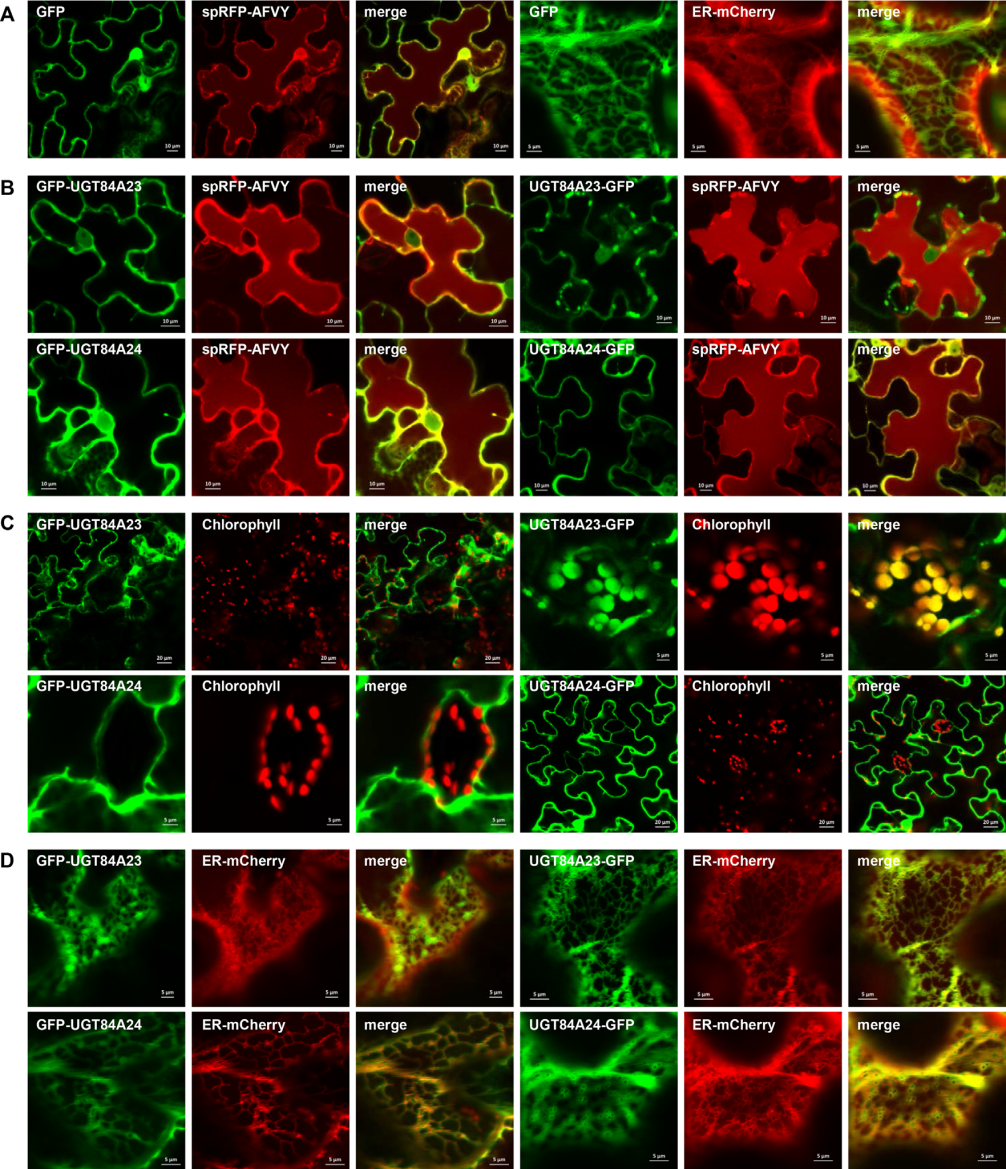
Subcellular localization of pomegranate UGT84A23 and UGT84A24 in *Nicotiana benthamiana* leaves analyzed by confocal microscopy. Green fluorescent protein (GFP) was fused to the N- (denoted GFP-UGT84A23 or GFP-UGT84A24) or C-terminus (denoted UGT84A23-GFP or UGT84A24-GFP) of the UGT proteins. (A) The free GFP expression alone or merged with spRFP-AFVY or ER-mCherry. (B) The expression of the GFP fused UGT84A23 and UGT84A24 proteins alone or merged with spRFP-AFVY. (C) The expression of the GFP fused UGT84A23 and UGT84A24 proteins alone or merged with chlorophyll autofluorescence. (D) The expression of the GFP fused UGT84A23 and UGT84A24 proteins alone or merged with ER-mCherry.

To investigate the subcellular localization of UGT84A23 and UGT84A24, N- and C-terminal GFP fused UGTs were transiently expressed in pomegranate and *N*. *benthamiana* leaves. Since limited infection by *A*. *tumefaciens* and weak expression of GFP were observed in pomegranate leaves (data not shown), *N*. *benthamiana* leaves were used for agroinfiltration with the localization constructs, individually or together with an organelle marker (Fig 7). Considering that UGT84A23 and UGT84A24 were most active under acidic conditions in enzyme assays (S6 Fig), the localization of UGT84A23 and UGT84A24 was compared to a vacuolar lumen marker spRFP-AFVY [35] (Fig 7B). UGT84A23 and UGT84A24 did not co-localize with spRFP-AFVY and there was a high accumulation of the C-terminal GFP fusion of UGT84A23 (UGT84A23-GFP) in punctate structures (Fig 7B).

Since 5-dehydroshikimic acid, the precursor for gallic acid biosynthesis, is produced by the shikimate pathway commonly present in the plastid, we compared GFP signals from the tagged UGT84A23 and UGT84A24 proteins with chloroplast autofluorescence (Fig 7C). While GFP-UGT84A23, GFP-UGT84A24 and UGT84A24-GFP localized outside of the chloroplast, the UGT84A23-GFP signal overlapped with that of chlorophyll autofluorescence (Fig 7C). Potential “KVEL” (KDEL variant) ER retention signal peptides were identified at the C-terminal ends of UGT84A23 and UGT84A24 (KVELAA and KVELVA, respectively), which may suggest their localization to the ER [36]. The fluorescent signals of the GFP-tagged UGT84A23 and UGT84A24 proteins were compared with those of the ER-mCherry marker (Fig 7D). GFP-UGT84A23, GFP-UGT84A24 and UGT84A24-GFP exhibited a diffused green fluorescence pattern similar to that of free GFP (Fig 7A and 7D). However, UGT84A23-GFP showed a defined ER localization (Fig 7D).

**Fig 8:**
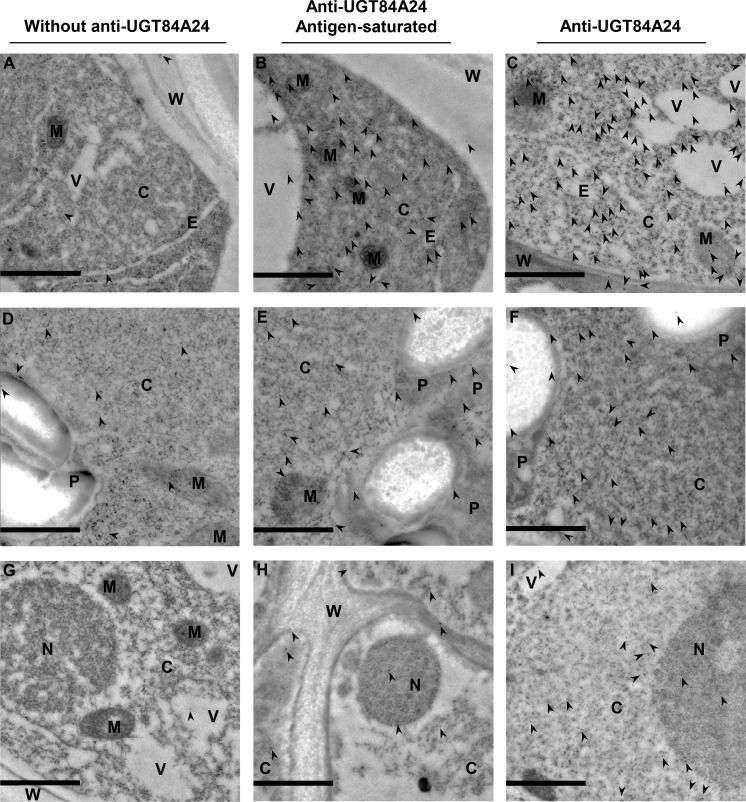
Subcellular localization of pomegranate UGT84A23 and UGT84A24 in root cells of pomegranate seedlings analyzed by immunogold electron microscopy. The grids were incubated with 1.5% goat serum only (A, D, G), anti-UGT84A24 saturated with UGT84A23 and UGT84A24 proteins (B, E, H), or anti-UGT84A24 (C, F, I). Representative 9 μm^2^ image areas encompassing various organelles are shown. Arrows indicate immunogold labels. C, cytosol; E, ER; V, vacuole; M, mitochondria; W, cell wall; P, plastid; N, nucleus. Scale bar, 1 nm.

A polyclonal antibody to UGT84A24 (anti-UGT84A24) was generated using recombinant UGT84A24 proteins and it cross-reacted to the highly identical (85%) UGT84A23 and UGT84A24 proteins (S10 Fig). To further investigate the subcellular localization of UGT84A23 and UGT84A24, immunogold labeling of the two UGTs was determined in roots of pomegranate seedlings using anti-UGT84A24 (Fig 8). Immunogold labels from the without anti-UGT84A24 and the antigen-saturated (mixture of UGT84A23 and UGT84A24 proteins) anti-UGT84A24 controls showed various levels of non-specific binding (Fig 8). However, binding of immunogold labels was significantly higher in grids that were hybridized with anti-UGT84A24 than the controls (Fig 8), which is supported by quantification of immunogold particles using a previously described method [29] (Table 2, *p* = 3 x 10^−7^ based on the ANOVA analysis and *p* < 1 x 10^−6^ based on the χ^2^ analysis). A total of seven subcellular compartments, including ER, vacuole, mitochondrium, cell wall, plastid, nucleus and cytosol, were defined on the electron micrographs (Fig 8). Of the seven compartments, only cytosol showed a significant localization of UGT84A23 and UGT84A24 proteins according to ANOVA (*p* = 3 x 10^−7^) and χ^2^ (*p* < 1 x 10^−6^) analyses (Table 2).

**Table 2:** Distribution of immunogold particles in root cells of pomegranate seedlings.

Compartment	Without anti-UGT84A24 (n = 12)	Anti-UGT84A24 Antigen-saturated (n = 46)	Anti-UGT84A24 (n = 22)	Anti-UGT84A24 vs. Antigen-saturated ANOVA *p* value	Anti-UGT84A24 (observed) vs. Antigen-saturated (expected)		
					χ^2^ value	df	*p* value
Cytosol	1.33 ± 1.63	17.8 ± 11.2	49.4 ± 21	3 x 10^−7^	56.3	1	< 1 x 10^−6^
Vacuole	0.58 ± 0.41	3.16 ± 1.73	5.37 ± 3.37	0.013	1.54	1	0.21
ER	0.08 ± 0.22	2.39 ± 1.95	2.91 ± 1.91	0.44	0.11	1	0.74
Mitochondrium	0.06 ± 0.19	1.62 ± 1.21	3.17 ± 2.62	0.07	1.48	1	0.22
Cell wall	0.36 ± 0.43	3.94 ± 2.7	3.2 ± 1.78	0.43	0.14	1	0.71
Plastid	0.39 ± 0.34	4.46 ± 0.89	3.34 ± 0.56	0.27	0.28	1	0.6
Nucleus	0.29 ± 0.41	2.52 ± 1.21	2.03 ± 0.41	0.64	0.09	1	0.76
Total	3.1 ± 1.84	35.6 ± 12	69.4 ± 21.6	3 x 10^−7^	60.4	6	< 1 x 10^−6^

The antigen mix includes recombinant UGT84A23 and UGT84A24 proteins. The average number and standard deviation of immunogold particles detected per 9 μm^2^ image area are indicated. n, number of electron micrographs analyzed; ANOVA, analysis of variance; df, degree of freedom.

## Discussion

### UGT84A23 and UGT84A24 are involved in HT biosynthesis in pomegranate

Being a high energy molecule, β-glucogallin serves as donor of the galloyl moiety for several groups of plant specialized metabolites, such as HTs and galloylated flavan-3-ols [7, 37]. Therefore, UGT catalyzed β-glucogallin formation not only provides substrates for the acyltransferase catalyzed reactions in HT biosynthesis, but also constitutes a control point for channeling carbons to the HT biosynthetic pathway. Of the four candidate PgUGTs examined, only UGT84A23 and UGT84A24 displayed *in vitro* enzymatic activities towards UDP-glucose and gallic acid by forming the galloyl glucose ester, β-glucogallin (Fig 3, S2 Fig). When compared to other β-glucogallin forming UGTs, UGT84A23 and UGT84A24, like *Vv*gGT1-3, showed comparable activities on a range of BA and cinnamic acid derivatives, while the tea UGT84A22 exhibited varied activities on these substrates and the oak UGT84A13 prefers BA derivatives over those of cinnamic acids [11–13] (Fig 4, Table 1). However, unlike *Vv*gGT1-3 that are inactive towards flavonoid substrates, UGT84A23 and UGT84A24 formed galloyl glucosides from several flavonoid molecules [11] (Fig 4).

*A*. *rhizogenes* induced hairy roots often mimic the biochemical capacities of wild type roots and have been successfully utilized for gene discovery and functional characterization in different plant species [38–40]. Considering the time, expense and technical challenges associated with transforming pomegranate plants, we previously established a hairy root system that shows similar HT and phenolic profiles as wild type roots for manipulation of candidate HT metabolic gene expression in pomegranate [18]. The candidate UGT genes identified from the pomegranate fruit peel transcriptome are also expressed in roots [18]. As described above in results and also discussed below, knocking down *UGT84A23* and *UGT84A24* expression simultaneously led to interesting metabolic changes in pomegranate hairy roots. However, it is worth noting that perturbations in hairy root metabolism may not reflect the metabolic events that take place in other pomegranate tissues and/or under different environmental and developmental conditions.

When *UGT84A23* or *UGT84A24* was overexpressed in pomegranate hairy roots, punicalagin and HT pathway intermediate accumulation was not significantly changed despite largely enhanced transgene expression in the respective *UGT84A23* or *UGT84A24* overexpression lines (Fig 5A, S10 Fig). This observation may be explained by post-transcriptional feedback regulation of *UGT84A23* and *UGT84A24*, which modified protein abundance and/or activity in the overexpression lines. Alternatively, pathway bottlenecks could exist either upstream or downstream of the UGT catalyzed reaction. Substrates of the UGTs could be insufficient/inaccessible or products downstream of the UGT catalyzed reaction, such as β-glucogallin, might not be effectively transformed to HTs due to limiting enzyme activities in downstream reactions. However, in the latter scenario, one would expect to observe an increased accumulation of β-glucogallin or HT pathway intermediates, which contradicts the similar phenolic profiles in the overexpression lines and the vector transformed controls (S9 Fig).

**Fig 9:**
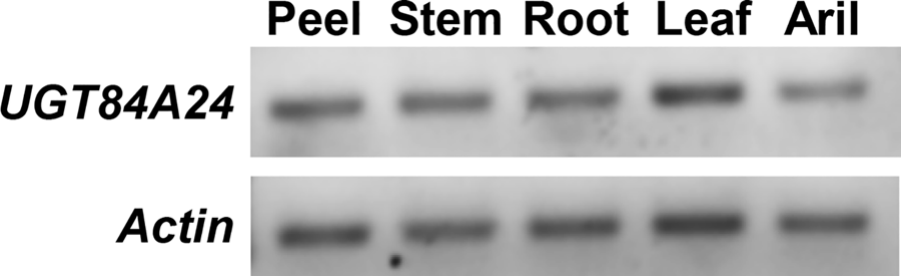
*UGT84A24* expression in pomegranate peel, stem, root, leaf and aril. Relative expression of *UGT84A24* and a pomegranate *Actin* gene [18] was determined by semi-quantitative RT-PCR.

Similar to the outcome of the overexpression studies, reduction of *UGT84A23* or *UGT84A24* expression did not result in significant changes in punicalagin and HT pathway intermediate accumulation in the respective knockdown hairy root lines (Fig 5B, S9 Fig). It could be that decreased gene expression in the single RNAi knockdown lines generated in this work was somehow not sufficient to achieve a significant reduction in punicalagins, though drastic reductions in *UGT84A24* expression were achieved for *UGT84A24* knockdown lines (Fig 5B). We also considered the possibility that reduced *UGT84A23* or *UGT84A24* transcript accumulation (and perhaps activities) in the single RNAi knockdown lines could be compensated for by another β-glucogallin forming activity. Since both UGT84A23 and UGT84A24 converted gallic acid and UDP glucose to β-glucogallin with similar specific activities (S4 and S6 Figs), we hypothesized that the two UGTs may have overlapping functions and could compensate for each other’s reduced activities in the single RNAi knockdown lines. Indeed, double knockdown of *UGT84A23* and *UGT84A24* in pomegranate hairy roots showed significant reductions in punicalagins, suggesting that both UGTs contribute to β-glucogallin production in hairy roots (Fig 6). In addition, the overexpression and the single RNAi knockdown lines exhibited phenolic profiles similar to the vector transformed controls (S9 Fig) and the double RNAi knockdown lines only affected the HT pathway related metabolites (Fig 6), suggesting that UGT84A23 and UGT84A24 may not be involved in the glycosylation of other phenolic acids and flavonoids in hairy roots despite their significant specificities towards these substrates in enzyme assays (Fig 4). *UGT84A23* expression was previously shown to correlate with punicalagin levels in various wild type pomegranate tissues [18]. On the other hand, *UGT84A24* exhibited a consistent expression profile across different pomegranate tissues (Fig 9). The distinct expression patterns of *UGT84A23* and *UGT84A24* suggested that they could be differentially regulated and may serve different roles in pomegranate.

### Subcellular localization of UGT84A23 and UGT84A24 suggests that β-glucogallin formation takes place in the cytosol

Isolation of β-glucogallin forming UGTs from pomegranate allowed further investigation of the subcellular localization of these proteins and their catalyzed reactions. Localization of GFP-UGT84A23, GFP-UGT84A24 and UGT84A24-GFP in *N*. *benthamiana* leaves indicated that UGT84A23 and UGT84A24 function outside of the vacuole, chloroplast and ER (Fig 7). The unusual expression patterns of UGT84A23-GFP in *N*. *benthamiana* leaves could be due to interference of the C-terminal GFP tag with proper folding or subcellular targeting of the protein (Fig 7). In line with the observations from the transient expression study, immunogold labeling indicated that UGT84A23 and UGT84A24 are localized in the cytosol in roots of pomegranate seedlings (Fig 8, Table 2). The cytosolic localization of UGT84A23 and UGT84A24 is consistent with those of previously characterized UGTs in several plant specialized metabolic pathways, though the glycosylated products and/or the downstream reactions are often found in other subcellular compartments [41–44].

For β-glucogallin production, UGT84A23 and UGT84A24 require gallic acid as a substrate, which is synthesized from 5-dehydroshikimic acid of the shikimate pathway [45]. Plastidic localization of the shikimate pathway has been well established [46, 47]. Experimental evidence from *N*. *tobacum*, *N*. *sylvestris* and *Pisum sativum* also supports a duplicated shikimate pathway in the cytosol [48–51]. In addition, multiple copies of the bifunctional enzyme 5-dehydroquinate dehydratase/shikimate dehydrogenase (DHQD/SDH), which leads to 5-dehydroshikimc acid, shikimic acid and gallic acid formation, were found in several woody species, such as *Fragria vesca*, *V*. *vinifera* and *Theobroma cacao* [46]. It is conceivable that gallic acid synthesized by the cytosolic DHQD/SDH isozyme could be supplied to UGT84A23 and UGT84A24 catalyzed reactions. On the other hand, if gallic acid is synthesized by the plastidic DHQD/SDH isozyme, it may require transport out of the plastid into the cytosol. The *DHQD/SDH* gene(s) remain to be identified and characterized in pomegranate.

### Galloyl glucoside production constitutes shunt reactions for gallic acid utilization in pomegranate hairy roots

**Fig 10:**
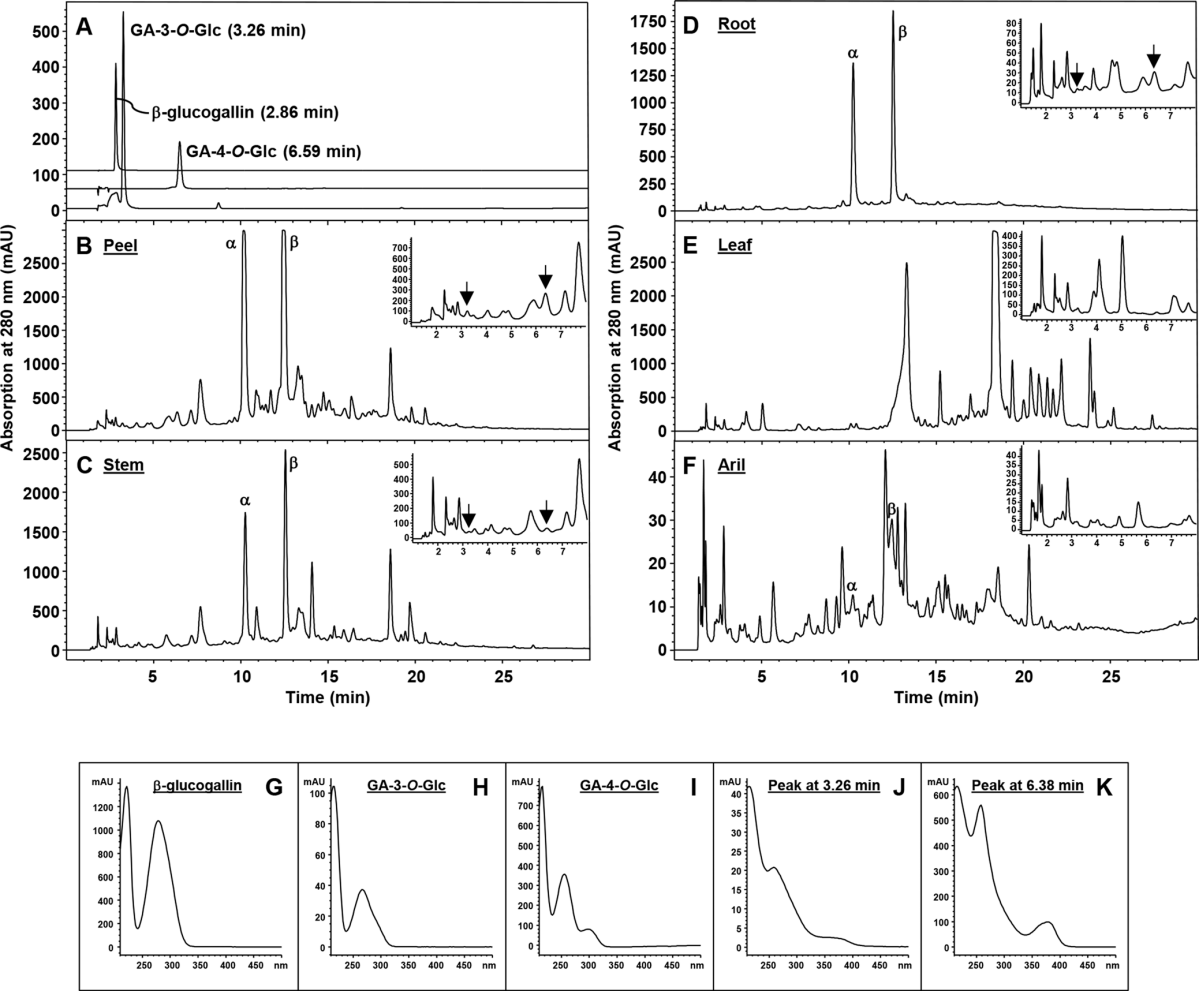
Phenolic metabolites from different pomegranate tissues. (A) β-glucogallin, gallic acid-3-*O*-β-D-glucopyranoside (GA-3-*O*-Glc) and gallic acid-4-*O*-β-D-glucopyranoside (GA-4-*O*-Glc) standards. HPLC elution profiles of phenolic metabolites from fruit peel (B), stem (C), root (D), leaf (E), and aril (F), as well as absorption spectra of β-glucogallin (G), GA-3-*O*-Glc (H), and GA-4-*O*-Glc (I) are shown. Metabolites eluted from 1 min to 8 min are shown as an inset in the corresponding panel. The peaks eluted at 3.26 min (the same retention time as that of GA-3-*O*-Glc, but with a different absorption spectrum) and 6.38 min (a similar retention time as that of GA-4-*O*-Glc, but with a different absorption spectrum) in peel, stem and root tissues are indicated with arrows and their absorption spectra are shown in (J) and (K), respectively. These two peaks were not detected in extracts obtained from leaf and aril tissues. α, punicalagin isomer α; β, punicalagin isomer β.

Accumulation of galloyl glucosides, including GA-3-*O*-Glc, GA-4-*O*-Glc and di-galloyl glucose (with at least one gallic acid group), in *UGT84A23* and *UGT84A24* double knockdown hairy root lines suggested that when β-glucogallin forming UGT activities were attenuated, a shunt reaction instead utilized gallic acid to form galloyl glucosides (Fig 6). A readily available pool of gallic acid resulted from reduced β-glucogallin production could be utilized for producing monogalloyl glucosides by a galloyl glucoside forming UGT activity. As the four candidate PgUGTs examined did not form galloyl glucosides (Fig 3), the contributing UGT(s) remain elusive. Since the above-mentioned galloyl glucosides do not accumulate in wild type pomegranate tissues (Fig 10), the shunt reactions for gallic acid utilization could be a salvage mechanism by pomegranate to prevent high concentrations of gallic acid accumulation which may acidify the subcellular compartments. This is consistent with the notion that glycosylation and deglycosylation represents a mechanism for detoxification and maintaining homeostasis of small molecules in plants [52]. On the other hand, one cannot rule out the possibility that monogalloyl glucosides may possess *bona fide* biological functions and are produced by pomegranate plants only under particular conditions. Additionally, the increased availability of gallic acid may initiate non-specific side reactions catalyzed by UGT(s) that serve other primary roles in pomegranate.

In conclusion, experimental evidence from enzyme assays and transgenic hairy root lines indicates that UGT84A23 and UGT84A24 contribute to a critical reaction of the HT biosynthetic pathway by producing the activated substrate β-glucogallin. The double RNAi knockdown and gene expression data also suggested that UGT84A23 and UGT84A24 possess overlapping yet potentially distinct activities. As compartmentalization of enzymes, substrates and products could play important roles in pathway dynamics and regulation, localization of UGT84A23 and UGT84A24 provides a first glimpse into a series of chemical reactions in the HT biosynthetic pathway. This study of UGT84A23 and UGT84A24 paves the way for understanding the biogenetic relationship and regulation of the HT and related biochemical pathways as well as manipulation of HT metabolism with the goal of improving plant, animal and human health.

## Supporting Information

S1 Fig *E*. *coli* expression and purification of pomegranate UGT proteins. Protein expression was induced with 0.5 mM isopropyl β-D-1-thiogalactopyranoside (IPTG) at 16°C overnight. The His-tagged recombinant proteins were purified using the MagneHis protein purification system. The protein samples were separated by SDS-PAGE and stained with Coomassie blue. M, protein molecular mass marker; U, total lysate from uninduced cells transformed with the pomegranate UGT construct; I, total lysate from cells transformed with the pomegranate UGT construct and induced by IPTG; S, supernatant of the pomegranate UGT-transformed and IPTG-induced cells; P, insoluble pellet of the pomegranate UGT-transformed and IPTG-induced cells; R, purified recombinant protein. The arrows indicate the recombinant proteins of expected sizes.
(TIF)

S2 Fig MS and MS/MS analyses of UGT84A23 and UGT84A24 enzyme assay products and galloyl glucose standards. Parent ion peak [M-H]^-^ (*m/z*) 331.07 (A) and daughter ion peaks (*m/z*) (B) of β-glucogallin, gallic acid-3-O-β-D-glucopyranoside and gallic acid-4-O-β-D-glucopyranoside standards as well as UGT84A23 and UGT84A24 enzyme assay products are shown.
(TIF)

S3 Fig Alkaline hydrolysis of UGT enzyme assay products and galloyl glucose standards. UGT enzyme assays were performed at 30°C for 1 h. The assay mixture or the authentic standards were then incubated with 0.2 N NaOH at 30°C for 5 min or 1 h. Alkaline hydrolysis products of the 5-min incubation are shown.
(TIF)

S4 Fig Effect of freezing and thawing on UGT84A23 and UGT84A24 activities. UGT enzyme assays were carried out using proteins that were either freshly prepared or previously frozen at -80°C and thawed with gallic acid and UDP-glucose as substrates. The specific activity (nkat/mg) of the proteins toward substrates was expressed as nmol of gallic acid substrate converted/s (nkat) by 1 mg of protein. Each data point represents the mean of three reactions ± SD.
(TIF)

S5 Fig Effect of reaction temperature on UGT84A23 and UGT84A24 activities. The UGT proteins were incubated with gallic acid and UDP-glucose at 0°C and 20°C—55°C with 5°C intervals, at pH 7. The specific activity (nkat/mg) of the proteins toward substrates was expressed as nmol of gallic acid substrate converted/s (nkat) by 1 mg of protein. Each data point represents the mean of three reactions ± SD.
(TIF)

S6 Fig Effect of reaction pH on UGT84A23 and UGT84A24 activities. The UGT proteins were incubated with UDP-glucose and gallic acid at pH 3.5–8, with 0.5 pH increments, at 30°C. The specific activity (nkat/mg) of the proteins toward substrates was expressed as nmol of gallic acid substrate converted/s (nkat) by 1 mg of protein. Each data point represents the mean of three reactions ± SD.
(TIF)

S7 Fig UGT84A23 activities towards selected phenolic acid and phenylpropanoid substrates. Enzyme assays and control reactions (no substrates, boiled protein) are shown. The enzyme assay products are indicated with arrows. BA, benzoic acid; 2-HBA, 2-hydroxybenzoic acid; 3-HBA, 3-hydroxybenzoic acid; 2,4-DHBA, 2,4-dihydroxybenzoic acid.
(TIF)

S8 Fig UGT84A24 activities towards selected phenolic acid and phenylpropanoid substrates. Enzyme assays and control reactions (no substrates, boiled protein) are shown. The enzyme assay products are indicated with arrows. BA, benzoic acid; 2-HBA, 2-hydroxybenzoic acid; 3-HBA, 3-hydroxybenzoic acid; 2,4-DHBA, 2,4-dihydroxybenzoic acid.
(TIF)

S9 Fig HPLC chromatograms for phenolic extracts of representative overexpression and RNAi knockdown hairy root lines of *UGT84A23* (A and C) or *UGT84A24* (B and D) overlaid with the respective vector transformed controls.
(TIF)

S10 Fig The anti-UGT84A24 polyclonal antibody cross-reacted to His-tagged UGT84A23 and UGT84A24 recombinant proteins purified from *E*. *coli*. The protein bands above 66 kDa were co-purified from *E*. *coli* lysates. M, protein molecular mass marker.
(TIF)

S1 Table Primers used for cloning of candidate pomegranate UGTs for *in vitro* enzyme assays and manipulation of gene expression in hairy roots. NS, no stop codon. Restriction enzyme recognition sites were engineered into the primers for cloning into the pET28a or pHIS8 vectors. UGT85K15, NdeI and HindIII; UGT84A23, BamHI and SalI; UGT73AL1, BamHI and SalI; UGT84A24, BamHI and SalI. The restriction enzyme recognition sites are underlined.
(PDF)

S2 Table Primers used for semi-quantitative and real-time qPCR analyses. UBC4, ubiquitin C4; PGK, phosphoglycerate kinase; GAPD, glyceraldehyde 3-phosphate dehydrogenase; TBP, TATA-binding protein. The subsequent en dash and number differentiate primers designed for different regions of the same gene.
(PDF)
