# Supplementary figures and images for: Two UGT84 Family Glycosyltransferases Catalyze a Critical Reaction of Hydrolyzable Tannin Biosynthesis in Pomegranate (Punica granatum)
Source: PLoS One. 2016 May 26;11(5):e0156319. doi: 10.1371/journal.pone.0156319 (PMC4882073; doi:10.1371/journal.pone.0156319)

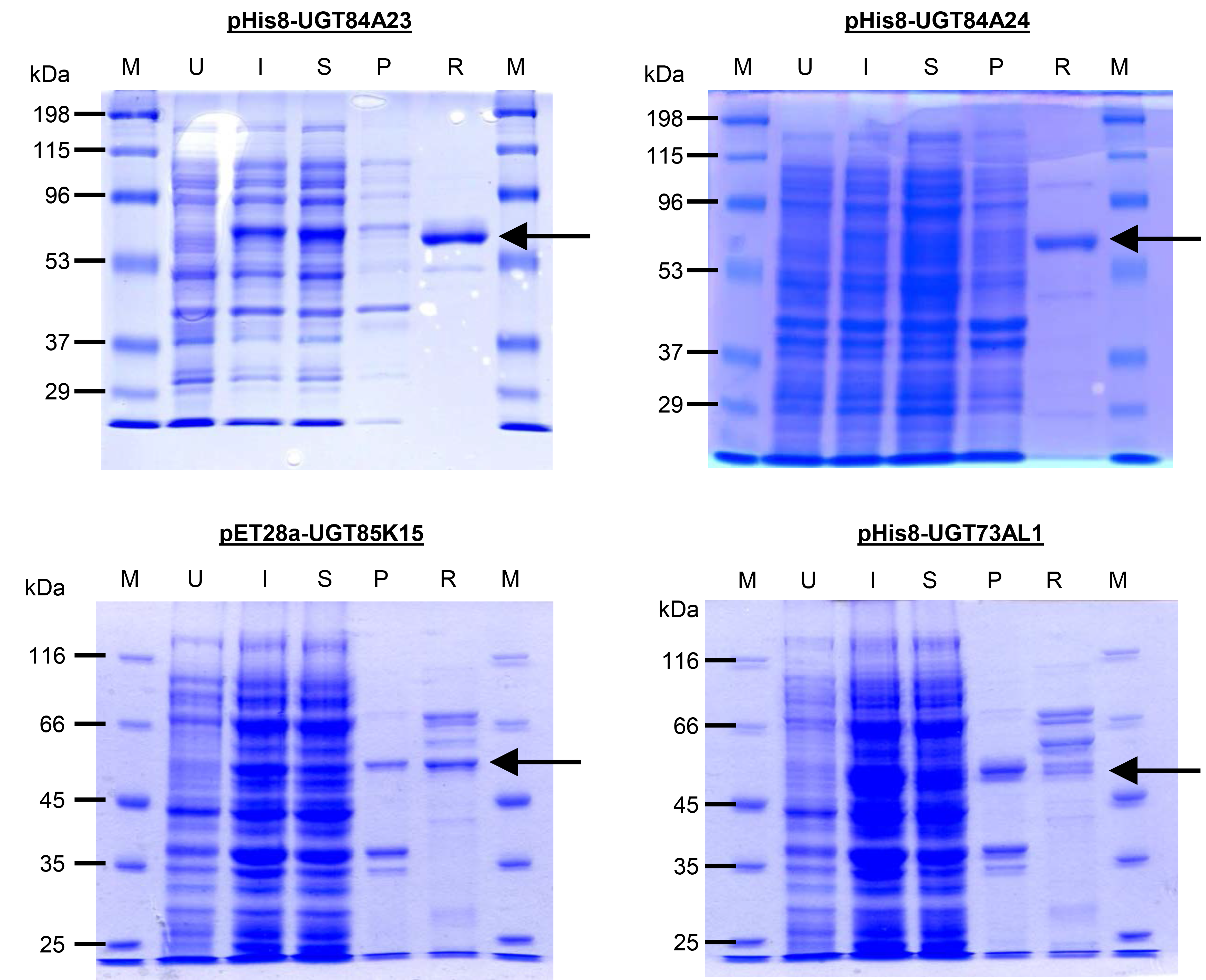

Supplement: S1 Fig — Protein expression was induced with 0.5 mM isopropyl β-D-1-thiogalactopyranoside (IPTG) at 16°C overnight. The His-tagged recombinant proteins were purified using the MagneHis protein purification system. The protein samples were separated by SDS-PAGE and stained with Coomassie blue. M, protein molecular mass marker; U, total lysate from uninduced cells transformed with the pomegranate UGT construct; I, total lysate from cells transformed with the pomegranate UGT construct and induced by IPTG; S, supernatant of the pomegranate UGT-transformed and IPTG-induced cells; P, insoluble pellet of the pomegranate UGT-transformed and IPTG-induced cells; R, purified recombinant protein. The arrows indicate the recombinant proteins of expected sizes. (TIF) [file pone.0156319.s001.tif]

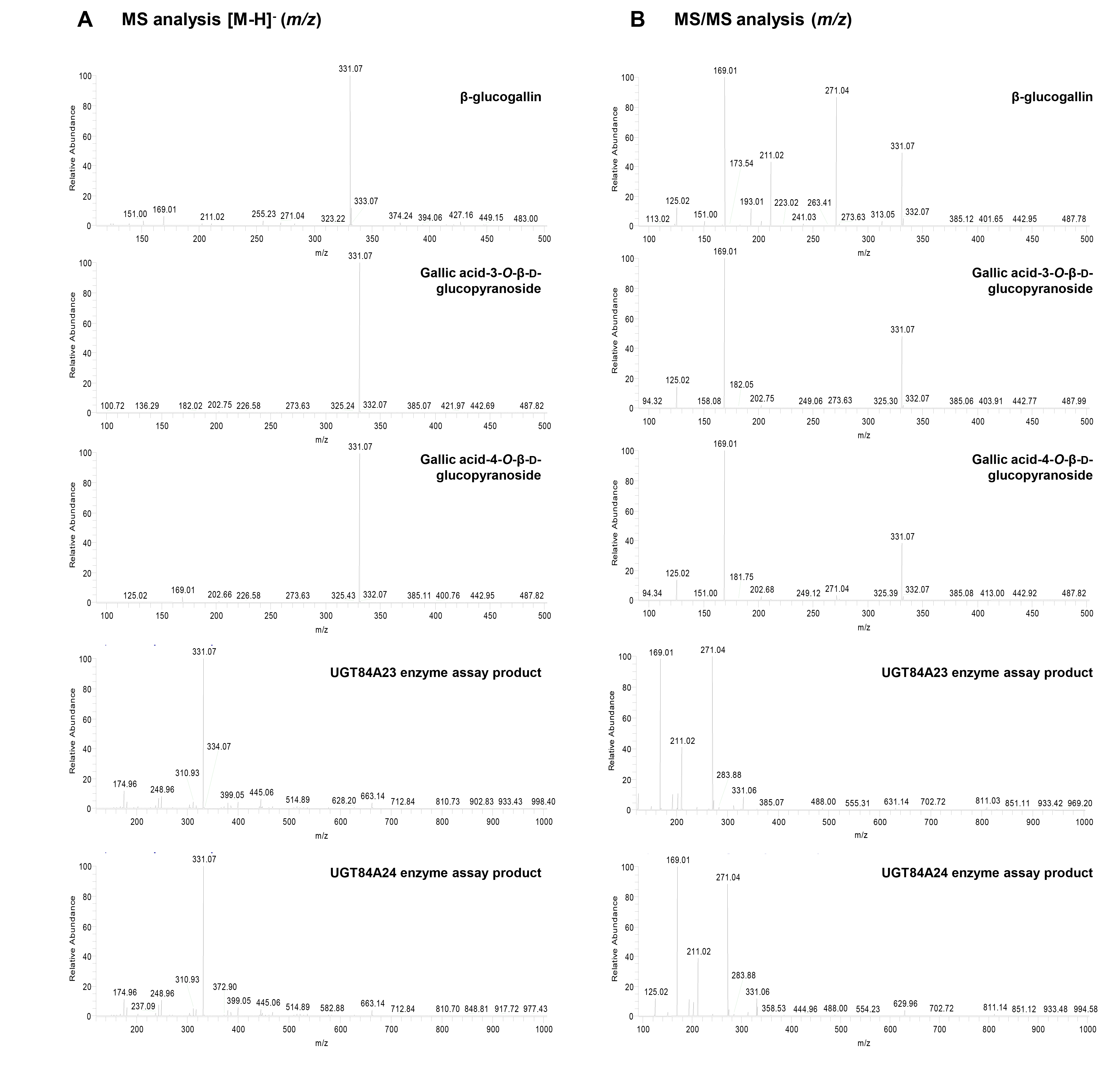

Supplement: S2 Fig — Parent ion peak [M-H]- (m/z) 331.07 (A) and daughter ion peaks (m/z) (B) of β-glucogallin, gallic acid-3-O-β-D-glucopyranoside and gallic acid-4-O-β-D-glucopyranoside standards as well as UGT84A23 and UGT84A24 enzyme assay products are shown. (TIF) [file pone.0156319.s002.tif]

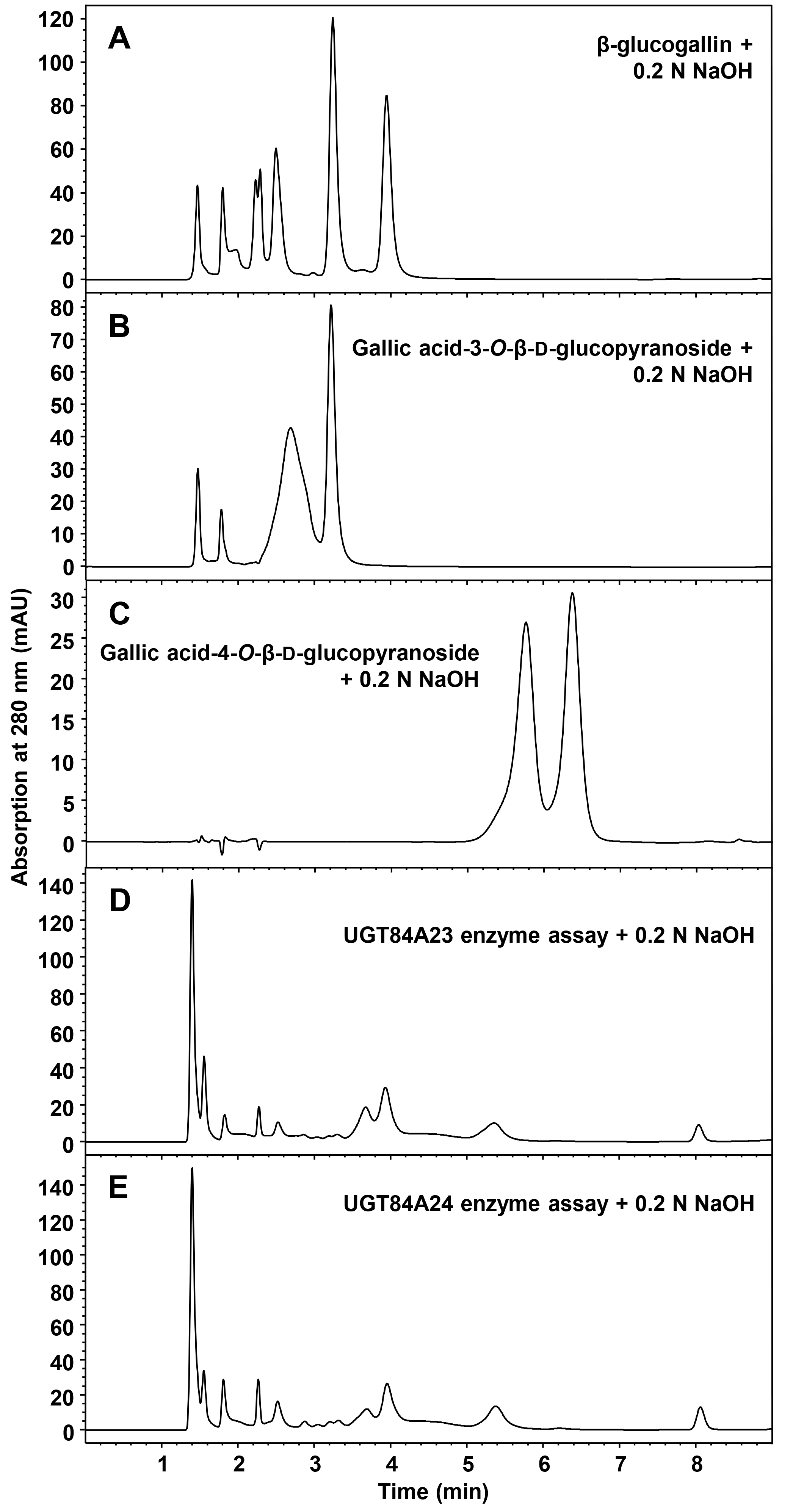

Supplement: S3 Fig — UGT enzyme assays were performed at 30°C for 1 h. The assay mixture or the authentic standards were then incubated with 0.2 N NaOH at 30°C for 5 min or 1 h. Alkaline hydrolysis products of the 5-min incubation are shown. (TIF) [file pone.0156319.s003.tif]

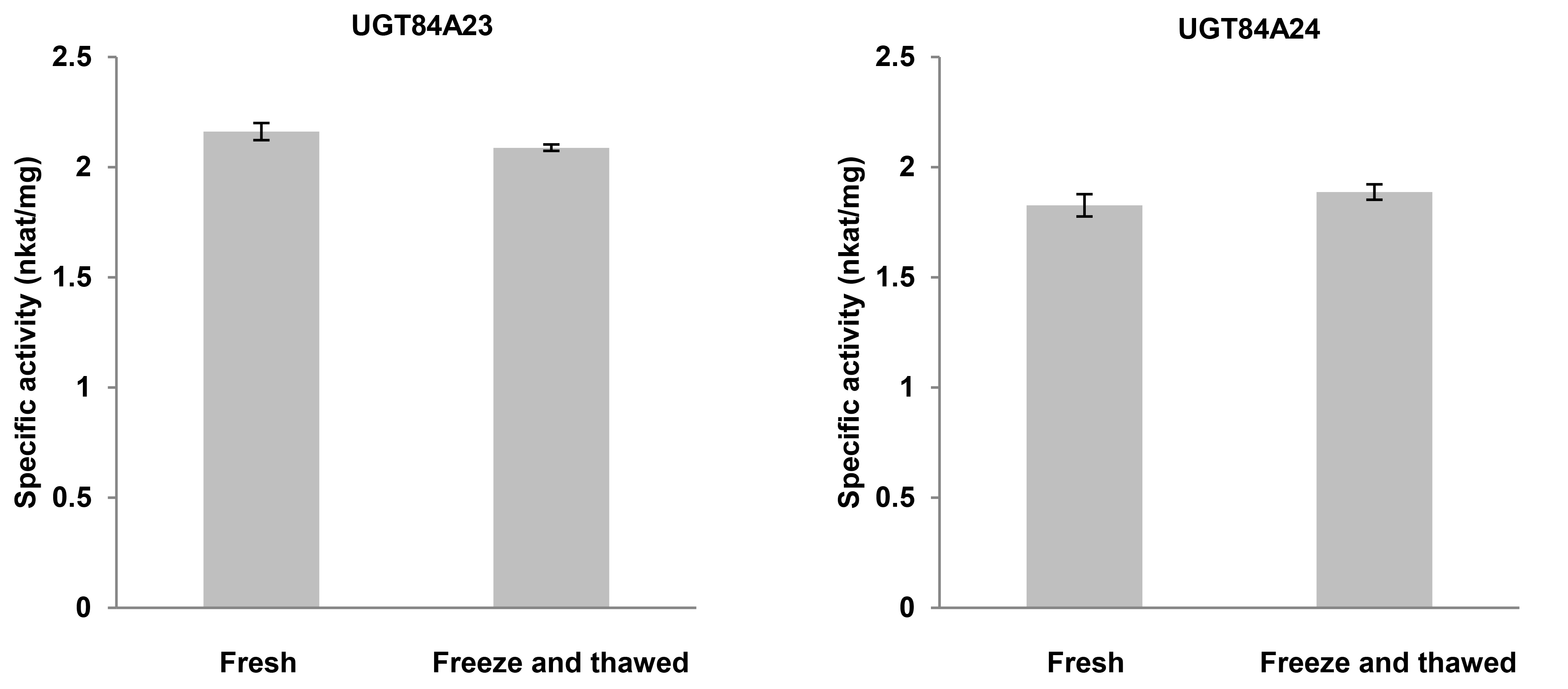

Supplement: S4 Fig — UGT enzyme assays were carried out using proteins that were either freshly prepared or previously frozen at -80°C and thawed with gallic acid and UDP-glucose as substrates. The specific activity (nkat/mg) of the proteins toward substrates was expressed as nmol of gallic acid substrate converted/s (nkat) by 1 mg of protein. Each data point represents the mean of three reactions ± SD. (TIF) [file pone.0156319.s004.tif]

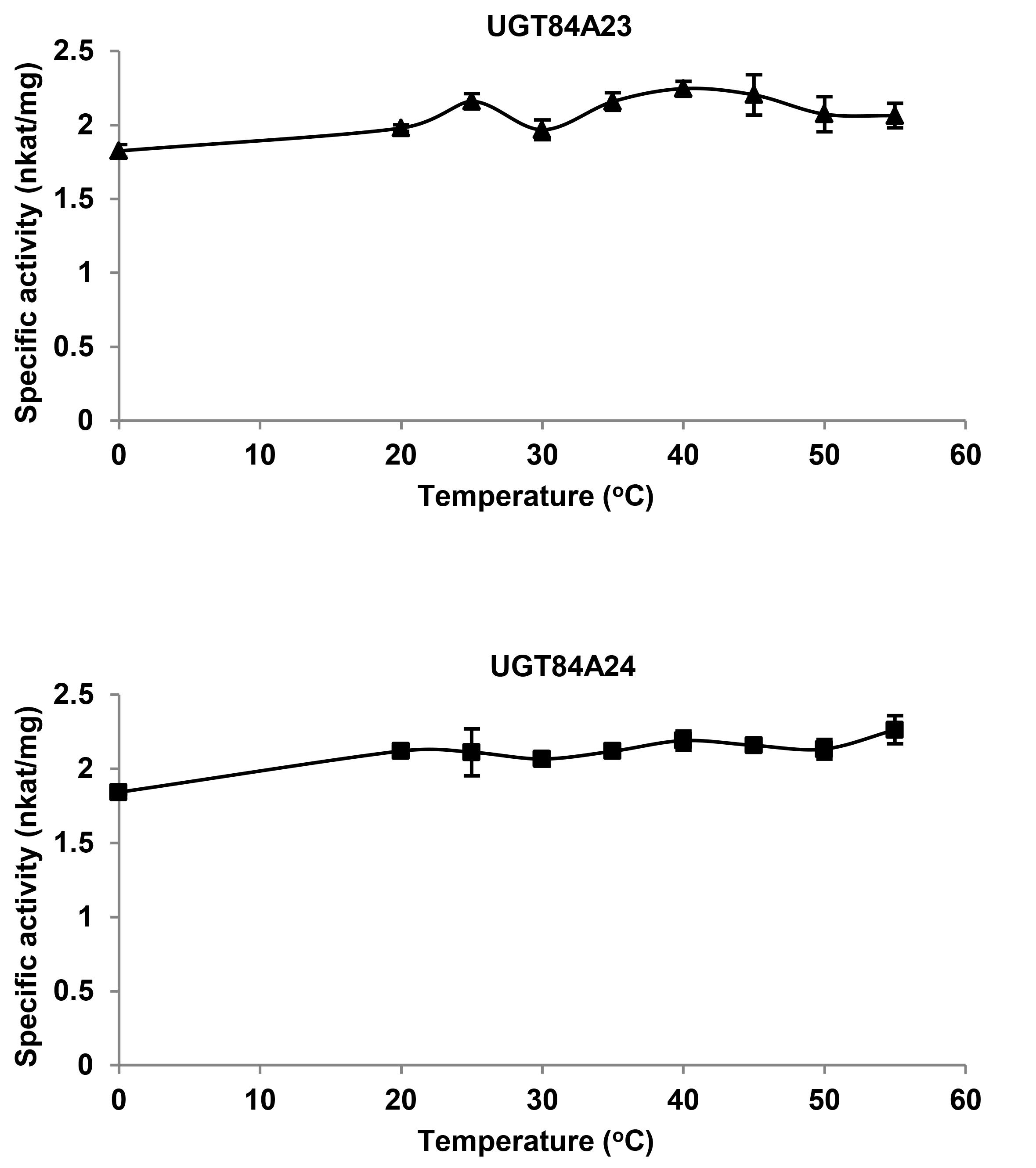

Supplement: S5 Fig — The UGT proteins were incubated with gallic acid and UDP-glucose at 0°C and 20°C—55°C with 5°C intervals, at pH 7. The specific activity (nkat/mg) of the proteins toward substrates was expressed as nmol of gallic acid substrate converted/s (nkat) by 1 mg of protein. Each data point represents the mean of three reactions ± SD. (TIF) [file pone.0156319.s005.tif]

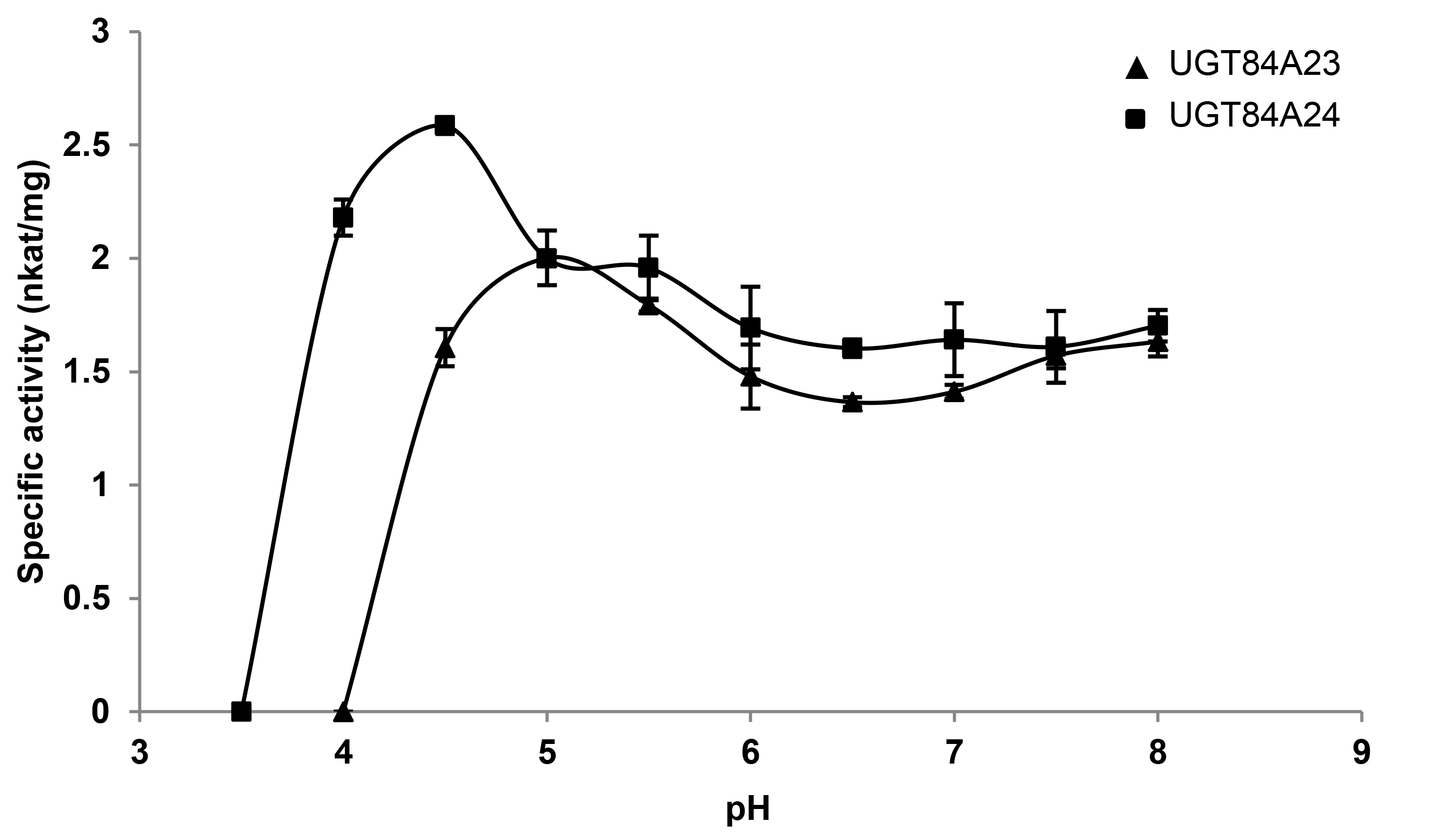

Supplement: S6 Fig — The UGT proteins were incubated with UDP-glucose and gallic acid at pH 3.5–8, with 0.5 pH increments, at 30°C. The specific activity (nkat/mg) of the proteins toward substrates was expressed as nmol of gallic acid substrate converted/s (nkat) by 1 mg of protein. Each data point represents the mean of three reactions ± SD. (TIF) [file pone.0156319.s006.tif]

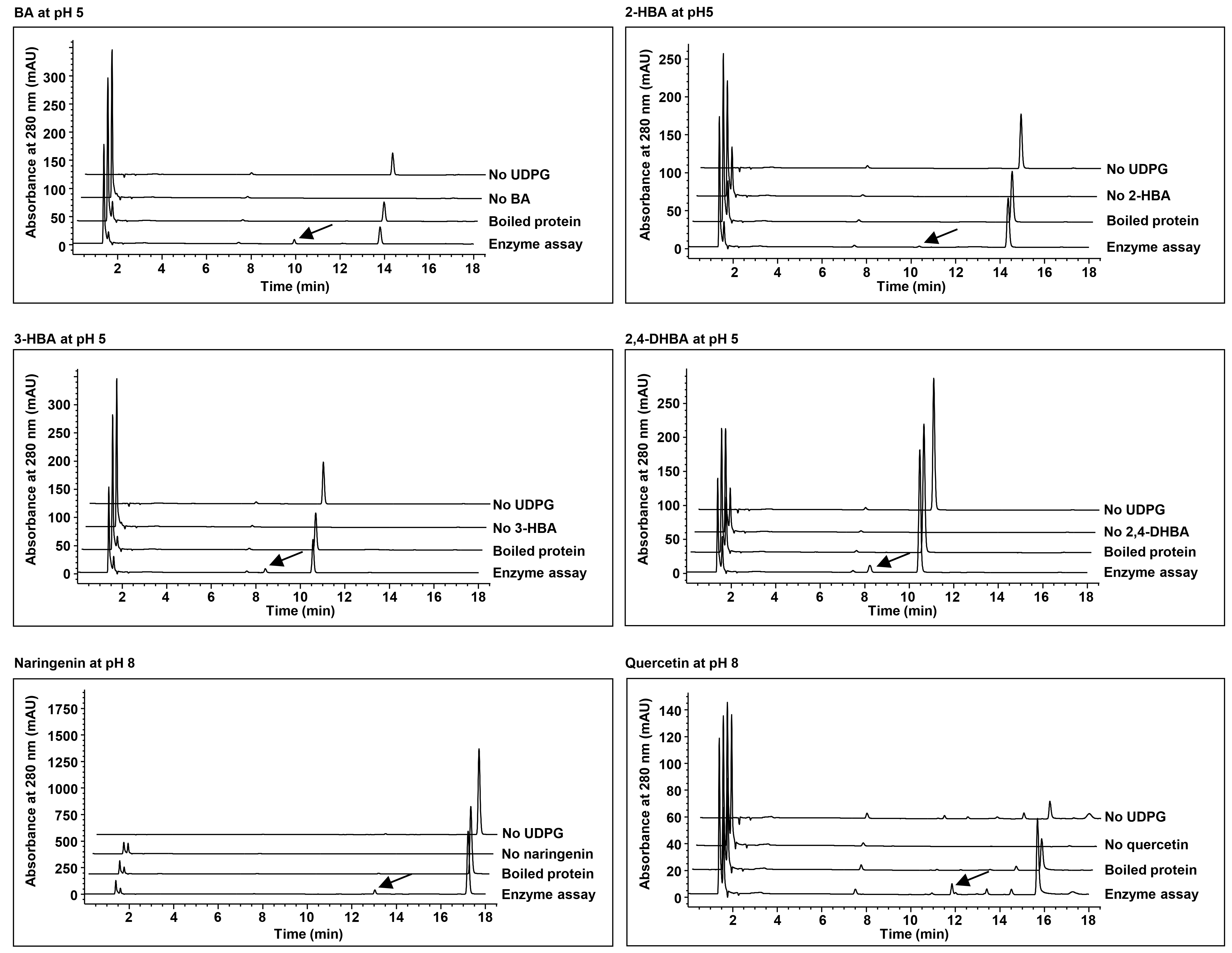

Supplement: S7 Fig — Enzyme assays and control reactions (no substrates, boiled protein) are shown. The enzyme assay products are indicated with arrows. BA, benzoic acid; 2-HBA, 2-hydroxybenzoic acid; 3-HBA, 3-hydroxybenzoic acid; 2,4-DHBA, 2,4-dihydroxybenzoic acid. (TIF) [file pone.0156319.s007.tif]

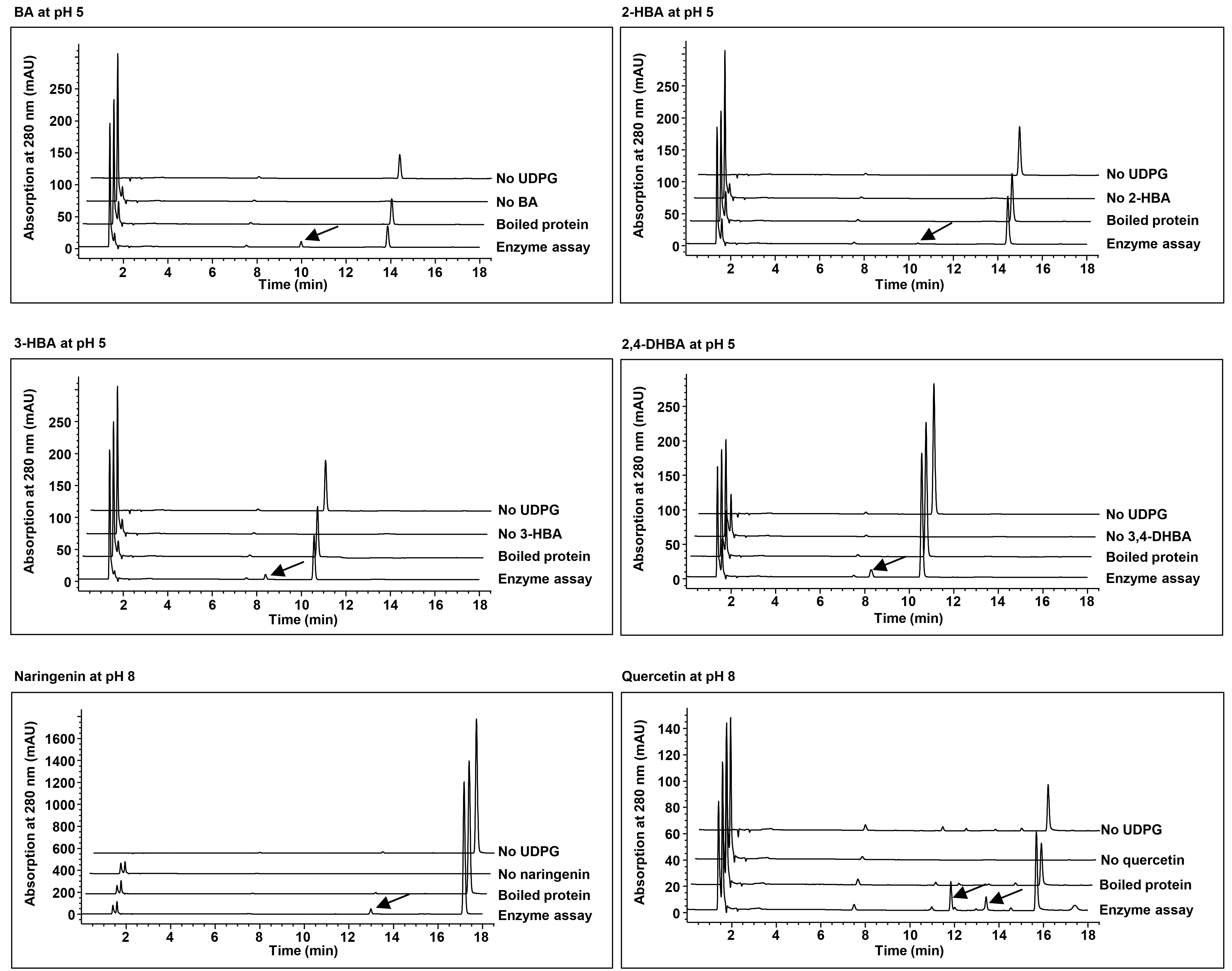

Supplement: S8 Fig — Enzyme assays and control reactions (no substrates, boiled protein) are shown. The enzyme assay products are indicated with arrows. BA, benzoic acid; 2-HBA, 2-hydroxybenzoic acid; 3-HBA, 3-hydroxybenzoic acid; 2,4-DHBA, 2,4-dihydroxybenzoic acid. (TIF) [file pone.0156319.s008.tif]

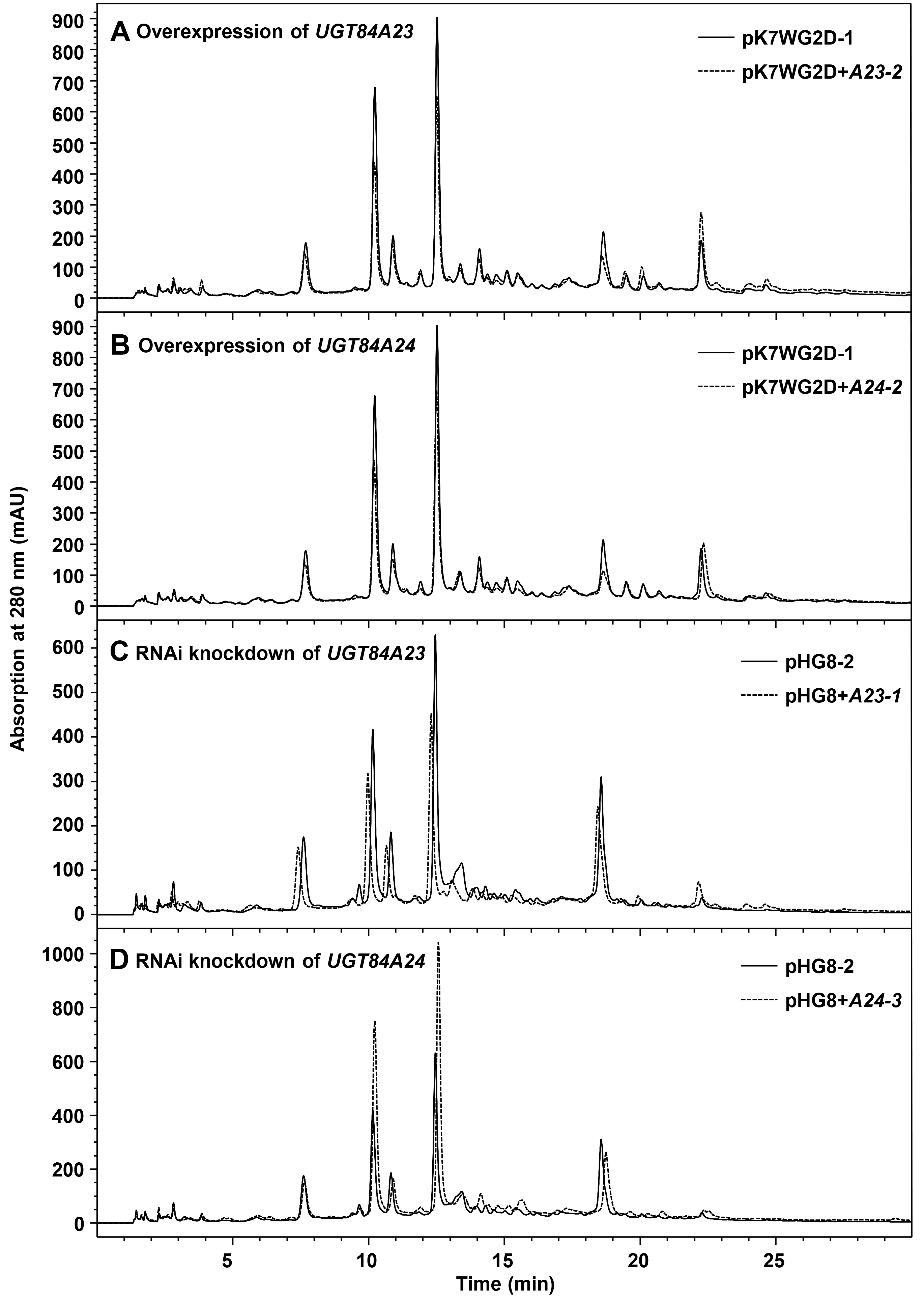

Supplement: S9 Fig — HPLC chromatograms for phenolic extracts of representative overexpression and RNAi knockdown hairy root lines of UGT84A23 (A and C) or UGT84A24 (B and D) overlaid with the respective vector transformed controls. (TIF) [file pone.0156319.s009.tif]

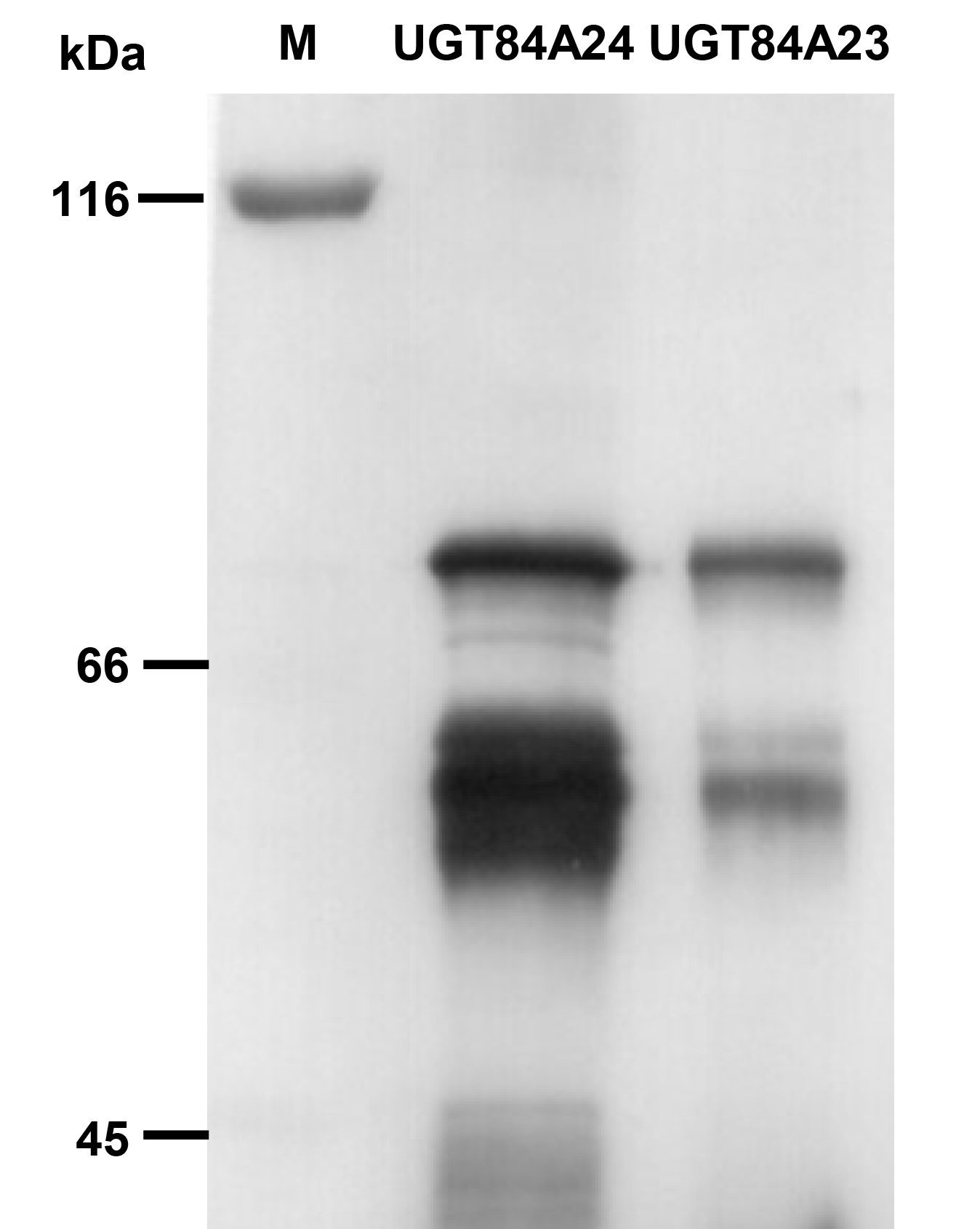

Supplement: S10 Fig — The protein bands above 66 kDa were co-purified from E. coli lysates. M, protein molecular mass marker. (TIF) [file pone.0156319.s010.tif]
