# Supplementary material for: Two UGT84 Family Glycosyltransferases Catalyze a Critical Reaction of Hydrolyzable Tannin Biosynthesis in Pomegranate (Punica granatum)
Source: PLoS One. 2016 May 26;11(5):e0156319. doi: 10.1371/journal.pone.0156319 (PMC4882073; doi:10.1371/journal.pone.0156319)
Supplement: S1 Table — NS, no stop codon. Restriction enzyme recognition sites were engineered into the primers for cloning into the pET28a or pHIS8 vectors. UGT85K15, NdeI and HindIII; UGT84A23, BamHI and SalI; UGT73AL1, BamHI and SalI; UGT84A24, BamHI and SalI. The restriction enzyme recognition sites are underlined. (PDF) [file pone.0156319.s011.pdf]

Gene/Construct	Vector	Forward	Reverse
UGT85K15	pET28a	CATATGGACCCCGCTCCTCGTA	AAGCTTGCTAATATATATCATATCCCTC
UGT84A23	pHis8	CATGGCGGATCCATGGGTTCGGAGTCGTC	AGCTTGTCGACGTCATGCGGCGAGCTCTACCTT
UGT73AL1	pHis8	GGATCCATGGCCAATGAAGGTGAGAC	GTCGACTCAGCTTTCGAGCAAAGCC
UGT84A24	pHis8	CATGGCGGATCCATGGGGTCCGAGTCG	AGCTTGTCGACGTCAAGCGACCAATTCAACC
UGT84A23	pENTR/D	CACCATGGGTTCGGAGTCGTCACTTG	TCATGCGGCGAGCTCTACCTTCCC
UGT84A23 (NS)	pENTR/D	CACCATGGGTTCGGAGTCGTCACTTG	TGCGGCGAGCTCTACCTTCCC
UGT84A24	pENTR/D	CACCATGGGGTCCGAGTCGTTGGTTCAT	TCAAGCGACCAATTCAACCTTCCCG
UGT84A24 (NS)	pENTR/D	CACCATGGGGTCCGAGTCGTTGGTTCAT	AGCGACCAATTCAACCTTCCCG
UGT84A23 RNAi	pENTR/D	CACCACCGGAACCTCCAGGCATTCGTGGA	GTAGGTCGATGACGATACGATCCTCC
UGT84A24 RNAi	pENTR/D	CACCCATCCAAGCGTTTGTTGATG	AACAACCTTGGGGGAAAATC
UGT84A23+UGT8	pENTR/D	F1: CACCATCGGGAAGCAGATGAGGA	R1: ATCTGCTTCCCAATGATACAGG
4A24 CDS RNAi		F2: TTGGGAAGCAGATGAGGAAG	R2: GGACACGGGACGGTTCTG
UGT84A23+UGT8	pENTR/D	F1: CACCAAGGCCAACGGGAAGGTA	R1: TAGCTCAGTAGGTCGATGACGA
4A24 3' UTR RNAi		F2: TGAGCTAATTGATGGGAAATAG	R2: ACTTATTCGTTATATCTAGACAAAATATCA
