# Supplementary material for: Two UGT84 Family Glycosyltransferases Catalyze a Critical Reaction of Hydrolyzable Tannin Biosynthesis in Pomegranate (Punica granatum)
Source: PLoS One. 2016 May 26;11(5):e0156319. doi: 10.1371/journal.pone.0156319 (PMC4882073; doi:10.1371/journal.pone.0156319)
Supplement: S2 Table — UBC4, ubiquitin C4; PGK, phosphoglycerate kinase; GAPD, glyceraldehyde 3-phosphate dehydrogenase; TBP, TATA-binding protein. The subsequent en dash and number differentiate primers designed for different regions of the same gene. (PDF) [file pone.0156319.s012.pdf]

Gene	Forward (5'-->3')	Reverse (5'--> 3')	Amplicon (bp)
UGT84A23	GTGGCTGGACTCGAAACCA	GAAGCCCATACGCGATCTCA	101
UGT84A24	GCCGAGAACAAGCTGATCATG	TCCACTTGAGCGCATTCTCC	103
UBC4-1	CCAATGGAACATGTCTTCTGCA	CGATGGCATCGAAGAGGATT	101
UBC4-2	GTCGCTTGCCAATGGAACAT	TCGAAGAGGATTTTGAAGGAGC	101
PGK-1	AGGGTAAGACCCTCCCTGGA	TGGTTTGCTAAACTGCACGG	102
PGK-2	GAGAAGGTCGGGCTTGCTG	AGAGCGAGGACTCCAGGGA	101
GAPD-1	CGGACCATTTCCCCTTCC	GGGCAGGTTTATGACAGTGCTC	110
GAPD-2	ACTCGGCACCAGCTCGTG	CACCATCATGTTGATGCTGTTG	101
TBP-1	ATCCTTGTGTGAGCCCAATCC	AGCCGTATAAGTCTCGTCTCGC	101
TBP-2	CCGAAGATTGTCCTCCTGATG	AGGATTGGGCTCACACAAGG	101
Cyclophilin-1	GTTCATGTGCCAAGGTGGTG	CGGTGTGCTTCCTGATGAAGT	104
Cyclophilin-2	GCCAGCTGTCTTAAATGGTGC	AGGACCCAGACACCATAAGCA	102
Cyclophilin-3	AAGTGTTGTCAGGGATGGACG	GGGACTTCACCACTGTCTGCA	103
